# Cell-cycle-dependent regulation of DNA end resection by PLK1 and PLK3 without CtIP level modulation

**DOI:** 10.1016/j.isci.2026.116450

**Published:** 2026-07-01

**Authors:** Bing Pan, Fanghua Li, Emil Mladenov, Martin Stuschke, Beate Timmermann, George Iliakis

**Affiliations:** 1 Institute of Medical Radiation Biology, University Hospital Essen, University of Duisburg-Essen, Essen, Germany; 2 Division of Experimental Radiation Biology, Department of Radiation Therapy, University Hospital Essen, University of Duisburg-Essen, Essen, Germany; 3 Division of Hepatobiliary, Pancreas and Spleen Surgery, Department of General Surgery, Beijing Chao-Yang Hospital, Capital Medical University, Beijing, China; 4 Department of Particle Therapy, University Hospital Essen, West German Proton Therapy Centre Essen (WPE), West German Cancer Center (WTZ), German Cancer Consortium (DKTK), Essen, Germany; 5 Partner Site University Hospital Essen, German Cancer Research Center (DKFZ), Essen, Germany

**Keywords:** Molecular interaction, Molecular network, Cell biology, DSB repairs

## Abstract

DNA double-strand breaks (DSBs) are highly cytotoxic lesions repaired primarily by homologous recombination (HR) and non-homologous end joining (NHEJ), with alternative end joining (alt-EJ) and single-strand annealing (SSA) functioning as backup. Repair pathway choice is governed by DNA end resection, suppressing NHEJ and committing repair to homology-based processes. Using pharmacological inhibition and protein depletion approaches in irradiated human cells, we show that PLK1 alone controls resection in S-phase-irradiated cells, while PLK1 and PLK3 act redundantly in G_2_-phase-irradiated cells. This cell-cycle-dependent regulation operates through a mechanism distinct from SCF^SKP2^-APC/C^CDH1^-mediated CtIP level modulation. These findings establish PLK1 and PLK3 as cell-cycle-dependent regulators of DSB repair pathway choice, advancing our understanding of how cell-cycle progression is coupled to repair decisions through differential PLK activity.

## Highlights

• DNA end resection is regulated in a tight cell-cycle-dependent manner
• PLK1 inhibition impairs DNA end resection in S-phase-irradiated cells
• PLK1 and PLK3 redundantly regulate resection in G_2_-phase-irradiated cells
• PLK1 and PLK3 control resection distinct from SCF^SKP2^-APC/C^CDH1^ axis modulation

## Graphical abstract

**Figure:**
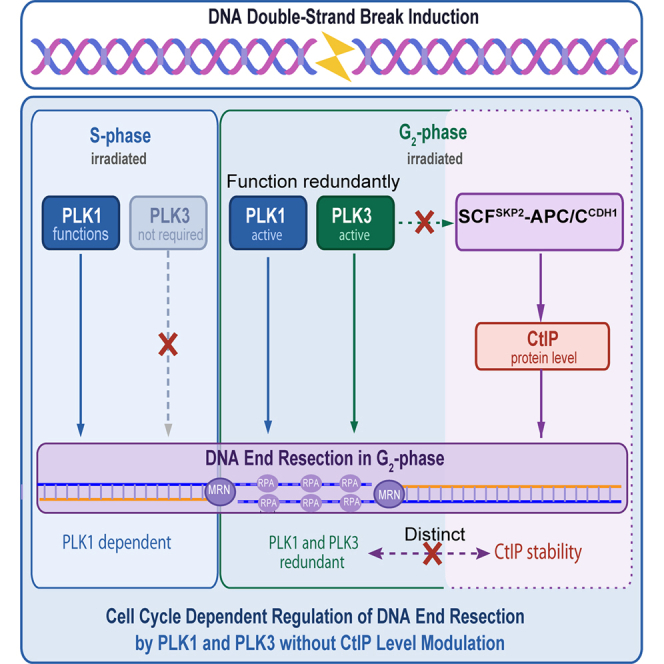

## Introduction

In higher eukaryotes, DNA double-strand breaks (DSBs) are primarily repaired by homologous recombination (HR) and non-homologous end joining (NHEJ). HR restores DNA with high fidelity using a sister chromatid as a template, restricting its activity to late S- and G_2_-phases.^1^ In contrast, NHEJ rejoins DNA ends rapidly and template-independently, functioning throughout the cell cycle. Although NHEJ often generates small insertions or deletions, its efficiency and speed make it the predominant DSB repair pathway, particularly following high-dose ionizing radiation (IR).^2^^,^^3^^,^^4^ When HR or NHEJ fails, more error-prone pathways such as alternative end joining (alt-EJ) and single-strand annealing (SSA) become active.^5^^,^^6^ Alt-EJ can generate chromosomal translocations linked to IR-induced malignancies.^7^^,^^8^ SSA aligns homologous repeats flanking DSBs, deleting the intervening sequence.^9^ HR and NHEJ preserve genome integrity, whereas alt-EJ and SSA maintain DNA continuity at the cost of some genomic stability.

How proper DSB repair pathways are selected remains unclear, but DNA end resection (hereafter resection) has emerged as a key determinant. Resection generates 3′ single-stranded DNA (ssDNA),^10^^,^^11^ inhibiting NHEJ and promoting homology-directed repair pathways, including HR, SSA, and partially alt-EJ.^12^ Resection occurs in two steps: initial resection, mediated by CtBP-interacting protein (CtIP) and the MRN complex (MRE11–RAD50–NBS1), generates short 3′-ssDNA, followed by extended resection by exonuclease 1 (EXO1) or DNA replication ATP-dependent helicase/nuclease 2 (DNA2)–Bloom syndrome protein (BLM) in the 5′–3′ direction, with the MRN complex contributing in the opposite direction.^13^ Resection is tightly cell-cycle-regulated, minimal in G_1_ and increasing in S/G_2_.^14^^,^^15^ Among other levels of regulation, this regulation is recently attributed to two E3-ubiquitin ligases controlling CtIP stability: the SKP1–CUL1–F-box protein^SKP2^ (SCF^SKP2^) complex, in which SKP2 is the S-phase-kinase-associated protein 2, and the anaphase-promoting complex/cyclosome with CDH1 (APC/C^CDH1^), in which CDH1 is the cell division cycle 20 homolog 1. Notably, SKP2 is a well-established substrate of APC/C^CDH1^, highlighting an active cross-talk between the SCF^SKP2^ and APC/C^CDH1^ complexes.^16^ The APC/C^CDH1^ is constitutively active in G_1_ and degrades CtIP, whereas SCF^SKP2^ acts as a positive regulator of resection in G_2_-phase cells by protecting CtIP from APC/C^CDH1^ complex-mediated degradation.^17^ Collectively, these findings indicate that the SCF^SKP2^-APC/C^CDH1^ axis dynamically regulates resection.^17^

Polo-like kinase 1 (PLK1) coordinates the interactions between SCF^β-TrCP^, another SCF E3 ubiquitin ligase in which β-TrCP is the beta-transducin repeat-containing protein, and APC/C^CDH1^. It not only activates APC/C^CDH1^ by promoting SCF^β-TrCP^-mediated degradation of APC/C inhibitor Emi1^18^ but also phosphorylates CDH1 to trigger its SCF^β-TrCP^-dependent degradation.^19^ Interestingly, PLK1 itself is an APC/C^CDH1^ substrate.^20^ In addition, SKP2 and β-TrCP are also linked through a regulatory feedback loop. F box and WD repeat domain-containing 2 (FBXW2), an E3 ligase substrate of β-TrCP, mediates SKP2 degradation.^21^ Thus, β-TrCP indirectly stabilizes SKP2. Conversely, SKP2 downregulation under energy restriction induces a CDK2-dependent increase in β-TrCP,^22^ establishing a feedback regulatory circuit. This network suggests that PLK1 may influence resection by modulating the SCF^SKP2^-APC/C^CDH1^ axis. Supporting this notion, SKP2 depletion causes a marked reduction in PLK1 protein level.^17^ However, it remains unclear whether this PLK1 downregulation is a cause or a consequence of APC/C^CDH1^ activation.

PLK1 has been reported to suppress resection in multiple ways. PLK1 inhibits the accumulation of breast cancer type 1 susceptibility protein (BRCA1) at DSB sites,^23^ phosphorylates MRE11 to reduce its recruitment to damaged DNA,^24^ and interacts with CtIP phosphorylated at serine 327 by cyclin-dependent kinase 1 (CDK1) and aurora kinase A (AuroraA), subsequently phosphorylating CtIP at serine 723. This PLK1-mediated modification inhibits CtIP’s ability to stimulate DNA2, thereby impairing extended resection.^25^^,^^26^ In contrast, PLK1 can also promote resection. It phosphorylates and inactivates peptidyl-prolyl *cis-trans* isomerase-like 2 (PPIL2), which ubiquitinates CtIP at lysine 426 to suppress resection, indirectly enhancing CtIP activity and resection level.^27^ These findings indicate that PLK1 may act as either a negative or a positive regulator of resection depending on the experimental context, highlighting the complexity of its function.

Another member of the Polo-like kinase family, Polo-like kinase 3 (PLK3), has also been implicated in regulating the DDR in G_1_ phase cells. PLK3 phosphorylates cell-division cycle 25A (Cdc25A) at serines 513 and 519, promoting its degradation. Consequently, PLK3 deficiency stabilizes Cdc25A and impairs the G_1_/S checkpoint caused by DNA damage.^28^ Additionally, PLK3 phosphorylates p53 at serine 20, preventing its proteasomal degradation and supporting IR-induced G_1_/S checkpoint activation.^29^ Additionally, PLK3 has also been shown to directly regulate resection by phosphorylating CtIP during DSB repair in G_1_-phase cells.^30^ Moreover, PLK3 promotes CDK2 activation by increasing cyclin E1 protein levels via a post-transcriptional mechanism, leading to suppression of APC/C^CDH1^ activity.^31^

In this study, we investigate the roles of PLK1 and PLK3 in regulating resection in cells exposed to IR during S- and G_2_-phases. We show that selective inhibition of PLK1 compromises G_2_-phase resection in S-phase-irradiated cells, while resection remains unaffected in cells irradiated and analyzed in G_2_-phase. In contrast, combined inhibition of PLK1 and PLK3 is required for suppression of resection in G_2_-phase-irradiated cells. Notably, PLK1 and PLK3 inhibition fails to activate APC/C^CDH1^ complex, suggesting a regulatory mechanism distinct from SCF^SKP2^-APC/C^CDH1^ module disruption. These findings reveal new layers of resection control by PLK1 and PLK3, highlighting cell-cycle-dependent regulatory processes.

## Results

### PLK1 inhibition suppresses G_2_-phase resection in S-phase-irradiated cells, but not in G_2_-phase-irradiated cells

We previously reported that SKP2 depletion impairs resection in cells irradiated during G_2_, but not in those irradiated in S-phase and subsequently entering G_2_.^17^ These findings highlight significant cell-cycle-dependent adaptations in resection regulation, which can only be detected and quantitatively measured using the specific analysis protocols established in our prior studies.^4^^,^^17^^,^^32^^,^^33^ Accordingly, the same protocols were employed here.

Cells are incubated with EdU for 30 min before IR to label S-phase cells at the time of IR. Chromatin-bound RPA70 is quantified as a surrogate of resection at various times post-IR. The analysis is restricted to G_2_-phase cells identified by PI staining. EdU staining is used to identify cells being in S-phase at the time of irradiation. Accordingly, EdU+ G_2_ cells reflect cells irradiated in S-phase that have progressed to G_2_, and EdU− G_2_ cells reflect cells irradiated in G_2_ and are analyzed in G_2_. Figure S1A shows the gating strategy for RPA70 quantification to estimate EdU+ and EdU− populations, and Figure S1B confirms that 10 Gy IR induces a robust RPA70 increase compared to unirradiated controls, both for cells irradiated in S-phase (EdU+) as well as for cells irradiated in G_2_-phase. This result validates the method for resection analysis in G_2_-phase cells and its ability to separate between cells irradiated in G_2_- and S-phases.

**Figure 1:**
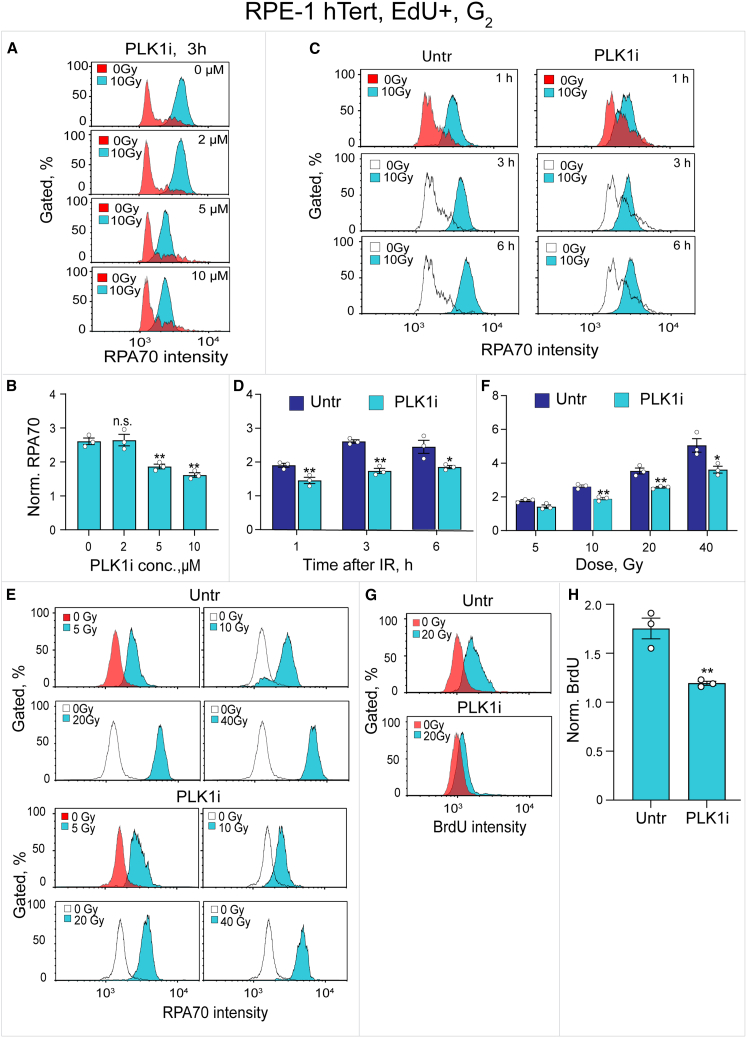
Inhibition of PLK1 with GSK461364 (PLK1i) suppresses resection in RPE-1 hTert cells irradiated during S-phase and analyzed in G_2_-phase Cells were pulse-labeled with EdU to mark cells in S-phase at the time of IR exposure and subsequently analyzed during G_2_-phase. RPA70 intensity, measured by flow cytometry, served as a readout for resection. (A) Representative flow cytometry plots showing RPA70 intensity in cells treated with increasing concentrations of PLK1i (0, 2, 5, or 10 μM), with or without 10 Gy IR. (B) Quantification of RPA70 intensity corresponding to (A). (C) Time course analysis of RPA70 accumulation at 1, 3, and 6 h post-10 Gy IR, in the presence or absence of 5 μM PLK1i. (D) Quantification of RPA70 intensity corresponding to (C). (E) RPA70 intensity across increasing IR doses (0, 5, 10, 20, and 40 Gy), comparing control and 5 μM PLK1i-treated cells. (F) Quantification of RPA70 intensity corresponding to (E). (G) Representative flow cytometry histograms showing ssDNA exposure detected by anti-BrdU antibody in cells pre-labeled with 10 μM BrdU for 24 h, followed by EdU labeling and 20 Gy IR (analyzed 3 h post-IR). (H) Quantification of BrdU intensity corresponding to (G). (A, C, E, and G) show representative flow cytometry plots from three independent experiments. Around 10,000 cells were analyzed per sample in each experiment. (B, D, F, and H) show quantification presented as mean ± SEM from *n* = 3 independent biological replicates. Statistical significance was assessed using an unpaired two-tailed Student’s *t* test. Significance is indicated as follows: ∗*p* < 0.05, ∗∗*p* < 0.01; and n.s., not significant.

GSK461364 is a potent, selective ATP-competitive PLK1 inhibitor with a reported Ki of approximately 2.2 nM and high selectivity over PLK2 and PLK3, exhibiting higher selectivity compared to other PLK1 inhibitors based on IC50 values, and has been widely used as a PLK1-targeting compound.^34^^,^^35^^,^^36^ To assess the role of PLK1 in resection, hTert-immortalized human retinal pigment epithelial (RPE-1 hTert) cells were pretreated for 1 h with GSK461364 (PLK1i) before exposure to 10 Gy IR and analyzed 3 h later. Figures 1A and 1B summarize the effects: PLK1i at 2 μM did not detectably suppress resection, whereas 5 μM and 10 μM significantly reduced resection in EdU+ G_2_-phase cells. Kinetic analysis (Figures 1C and 1D) showed that chromatin-bound RPA70 accumulated within 1 h post-IR and remained largely stable up to 6 h in EdU+ G_2_-phase cells in the absence of PLK1i. Similarly, in PLK1i-treated cells, resection was evident but reduced at all time points. These results indicate that PLK1 activity is required for efficient resection in EdU+ G2-phase cells, regardless of the duration after IR. All subsequent experiments therefore used 5 μM PLK1i with analysis at 3 h, unless indicated otherwise. Given that the DSB load significantly influences IR-induced DDR,^4^^,^^14^^,^^37^ we next exposed RPE-1 hTert cells to doses ranging from 5 Gy to 40 Gy. PLK1i significantly downregulated resection at IR doses ranging from 10 to 40 Gy (Figures 1E and 1F) and showed a consistent decrease even at 5 Gy, suggesting a dose-independent effect.

To validate these findings with an alternative method, we performed BrdU-based ssDNA detection. Cells were pre-labeled with 10 μM BrdU for 24 h prior to EdU labeling and irradiation (20 Gy, analyzed at 3 h post-IR). Consistent with the RPA70 assay, PLK1i markedly reduced the generation of ssDNA after irradiation, as measured by the accessibility of BrdU to the anti-BrdU antibody in EdU+ G_2_-phase cells, confirming that PLK1 activity is required for efficient resection in cells irradiated during S-phase and analyzed in G_2_ (Figures 1G and 1H).

**Figure 2:**
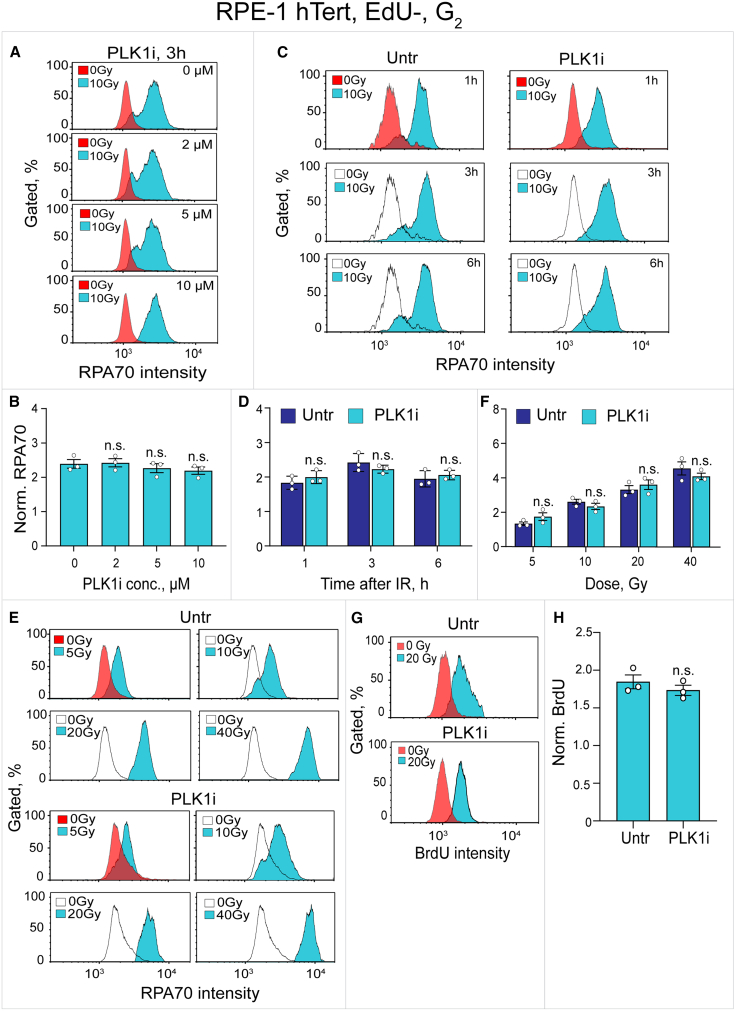
PLK1 inhibition with GSK461364 (PLK1i) fails to suppress resection in EdU− G_2_-phase RPE-1 hTert cells As in Figure 1, RPA70 or BrdU intensity was measured by flow cytometry to assess resection. In this case, EdU− G_2_-phase cells (irradiated and analyzed in G_2_) were examined. The experimental design and panel layout are identical to Figure 1. Unlike EdU+ cells, treatment with the PLK1i did not reduce RPA70 (A–F) or BrdU (G and H) intensity following irradiation. (A and B) Increasing concentrations of GSK461364 (0–10 μM) with or without 10 Gy IR. (C and D) Time course after 10 Gy IR (1, 3, and 6 h). (E and F) IR dose response (0–40 Gy). (G and H) BrdU-based ssDNA assay after 20 Gy IR (analyzed 3 h post-IR). (A, C, E, and G) show representative flow cytometry plots from three independent experiments. Around 10,000 cells were analyzed per sample in each experiment. (B, D, F, and H) show quantification presented as mean ± SEM from *n* = 3 independent biological replicates. Statistical analyses were performed using an unpaired two-tailed Student’s *t* test. Significance is indicated as follows: ∗*p* < 0.05, ∗∗*p* < 0.01; n.s., not significant.

Surprisingly, PLK1i (up to 10 μM) fails to downregulate resection in EdU− G_2_-phase RPE-1 hTert cells at any dose or time, measured by RPA70 detection or a BrdU-based assay (Figure 2), indicating that PLK1 regulates resection in G_2_-phase cells only when cells are irradiated during S-phase.

Similar results were observed in hTert-immortalized human fibroblast (82-6 hTert) cells: 5 μM PLK1i significantly suppressed resection in EdU+ G_2_-phase cells, with no further reduction at 10 μM (Figures S2A and S2B). Inhibition was consistent across IR doses (Figures S2C and S2D), and no effect was seen in EdU− G_2_-phase cells (Figure S3) indicating that PLK1-dependent, cell-cycle-specific regulation of resection is a general mechanism across cell lines.

### Combined PLK1 and PLK3 inhibition effectively suppresses resection in G_2_-phase-irradiated cells

**Figure 3:**
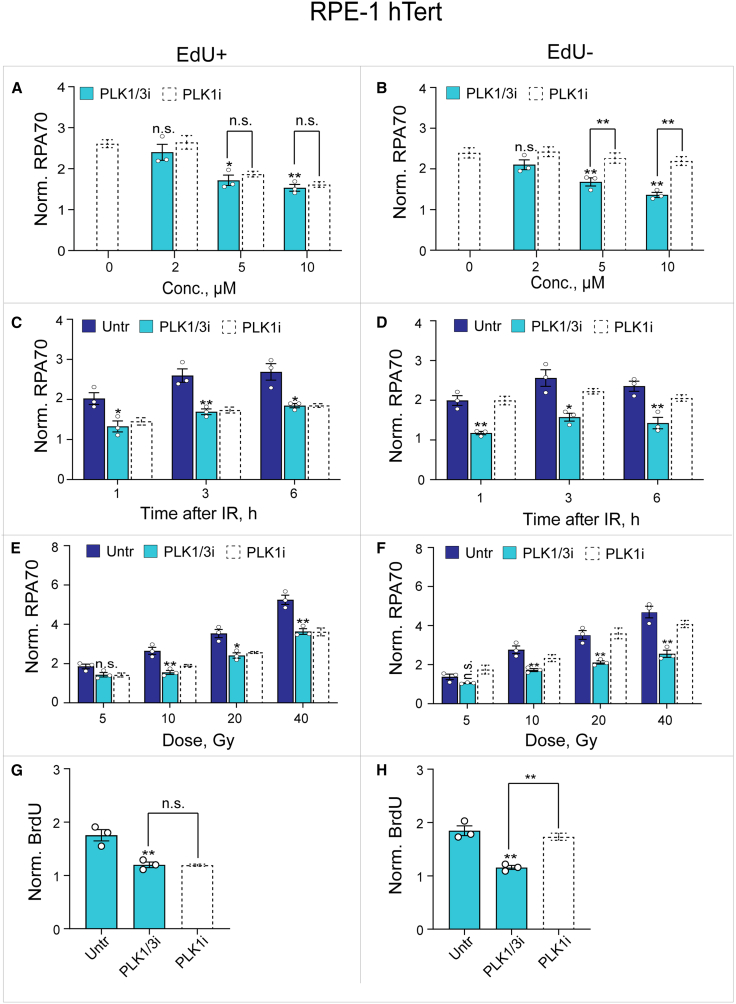
Effects of the PLK1/3 inhibitor GW843682X (PLK1/3i) on resection in EdU+ and EdU− G_2_-phase RPE-1 hTert cells (A) Effect of increasing concentrations of PLK1/3i (0, 2, 5, and 10 μM) on resection in EdU+ G_2_-phase cells. (B) Same as (A), but in EdU− G_2_-phase cells. (C) Time course analysis of RPA70 accumulation at 1, 3, and 6 h post-10 Gy IR, in the presence or absence of 5 μM PLK1/3i in EdU+ cells. (D) Same as (C), but in EdU− G_2_-phase cells. (E) RPA70 intensity across increasing IR doses (0, 5, 10, 20, and 40 Gy), comparing control and 5 μM PLK1/3i in EdU+ cells. (F) Same as (E), but in EdU− G_2_-phase cells. (G) BrdU-based ssDNA after 20 Gy IR (analyzed 3 h post IR) after treatment of 5 μM PLK1/3i in EdU+ G_2_ cells. (H) Same as (G), but in EdU− G_2_-phase cells. For (C–F), PLK1i and PLK1/3i experiments were performed independently, and the controls are not shared; comparisons between them are qualitative. For (A, B, G, and H), PLK1i and PLK1/3i experiments were performed together and share the same control, allowing quantitative comparisons. For comparison, data from PLK1i, previously shown in Figures 1 and 2, are included in the corresponding panels. To avoid overcrowding, PLK1i data are displayed as bars with dashed outlines. (A–H) show mean ± SEM from *n* = 3 independent biological replicates. Approximately 10,000 cells were analyzed per sample in each experiment. Statistical analyses were performed using an unpaired two-tailed Student’s *t* test. Significance is indicated as follows: ∗*p* < 0.05, ∗∗*p* < 0.01; n.s., not significant. Representative flow cytometry plots are shown in Figure S4.

PLK3 regulates resection in G_1_-phase cells by activating CtIP via T847 phosphorylation.^30^ We hypothesized that PLK3 may contribute to resection when PLK1 is inhibited and therefore tested the effects of combined PLK1/3 inhibition. GW843682X (PLK1/3i) is a selective ATP-competitive inhibitor against PLK1 and PLK3 with a similar potency and has been employed previously in studies investigating DDR.^30^^,^^32^^,^^38^^,^^39^ RPE-1 hTert cells were pretreated with 0–10 μM PLK1/3i for 1 h prior to 10 Gy IR, and resection was analyzed 3 h post-IR. PLK1/3i robustly suppressed resection in EdU+ G_2_-phase cells at 5–10 μM (Figures 3A and S4A). PLK1/3i also reduced resection in EdU− G_2_-phase cells, an effect absent when using PLK1i alone (Figures 3B and S4B). Kinetic analysis in both EdU+ and EdU− G_2_-phase cells (Figures 3C, 3D, S4C, and S4D) showed that PLK1/3i-mediated inhibition of resection was evident at all time points tested. Moreover, the suppression effects of PLK1/3i on resection were also independent of IR dose (Figures 3E, 3F, S4E, and S4F). In addition to RPA70 measurements, BrdU-based ssDNA detection at 3 h post-20 Gy IR confirmed that PLK1/3i suppresses resection in both EdU+ and EdU− G_2_-phase cells (Figures 3G, 3H, S4G, and S4H). Notably, Figures 3A–3C, 3E, and 3G show that PLK1/3i downregulates resection with similar effectiveness as PLK1i in EdU+ G_2_-phase cells, suggesting that PLK3 does not measurably contribute in S-phase-irradiated cells. It should be noted that the analyses in Figures 3C and 3E are qualitative, as PLK1i and PLK1/3i were assessed independently. In contrast, in Figures 3A and 3F, PLK1i and PLK1/3i were analyzed in parallel within the same experiment, allowing for direct statistical comparison.

Experiments in 82-6 hTert cells showed the similar results: PLK1/3i suppressed resection in both EdU+ and EdU− G_2_-phase cells across all IR doses tested (Figure S5). These findings indicate that dual PLK1 and PLK3 inhibition effectively blocks resection in G_2_-phase-irradiated cells and reveal a distinct, phase-specific contribution of PLK3 to resection regulation: it does not contribute in S-phase-irradiated cells but acts redundantly with PLK1 in cells irradiated in G_2_-phase.

### Suppression of resection in G_2_-phase-irradiated cells requires combined deactivation of PLK1 and PLK3

**Figure 4:**
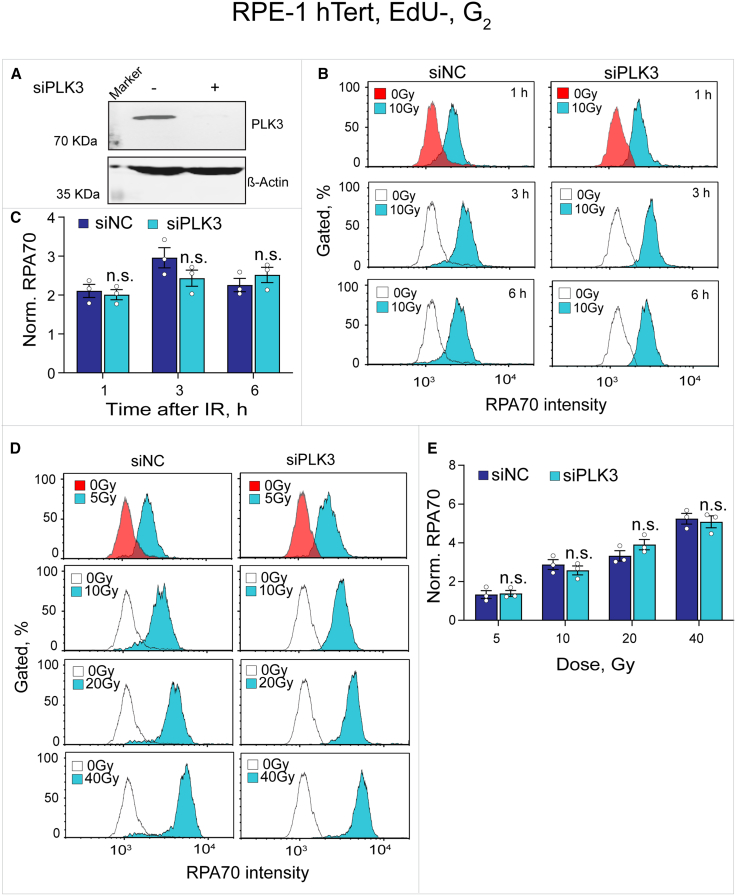
Effects of PLK3 depletion via RNA interference on resection in EdU− G_2_-phase RPE-1 hTert cells RPE-1 hTert cells were transfected with siRNA targeting PLK3. (A) Western blot confirming efficient PLK3 depletion, with samples collected 48 h after siRNA delivery. Experiments were repeated three times, and a representative blot is shown. This blot corresponds to the experiments shown in (B and C). (B) Time course analysis of RPA70 accumulation at 1, 3, and 6 h post-10 Gy IR, comparing cells transfected with control or PLK3-targeting siRNA. (C) Quantification of RPA70 intensity corresponding to (B). (D) RPA70 intensity across increasing IR doses (0, 5, 10, 20, and 40 Gy), comparing control and PLK3-depleted cells. (E) Quantification of RPA70 intensity corresponding to (D). (B and D) show representative flow cytometry plots from three independent experiments. Around 10,000 cells were analyzed per sample in each experiment. (C and E) show mean ± SEM from *n* = 3 independent biological replicates. Statistical analyses were performed using an unpaired two-tailed Student’s *t* test. Significance is indicated as follows: ∗*p* < 0.05, ∗∗*p* < 0.01; n.s., not significant.

PLK1 inhibition alone fails to alter resection levels in EdU− G_2_-phase cells, whereas PLK1/3i successfully reduces it. However, it was unclear whether PLK3 inhibition alone is sufficient or combined inhibition of PLK1 and PLK3 is necessary to suppress resection in G_2_-phase-irradiated cells. We therefore analyzed resection after PLK3 depletion using a specific siRNA. PLK3 depletion (Figure 4A) in RPE-1 hTert cells fails to alter resection in EdU− G_2_ phase cells up to 6 h after 10 Gy IR (Figures 4B and 4C) or at doses of 5–40 Gy (Figures 4D and 4E). These results indicate that PLK3 depletion alone cannot suppress resection and suggest that in G_2_-phase, PLK1 and PLK3 redundantly regulate resection.

**Figure 5:**
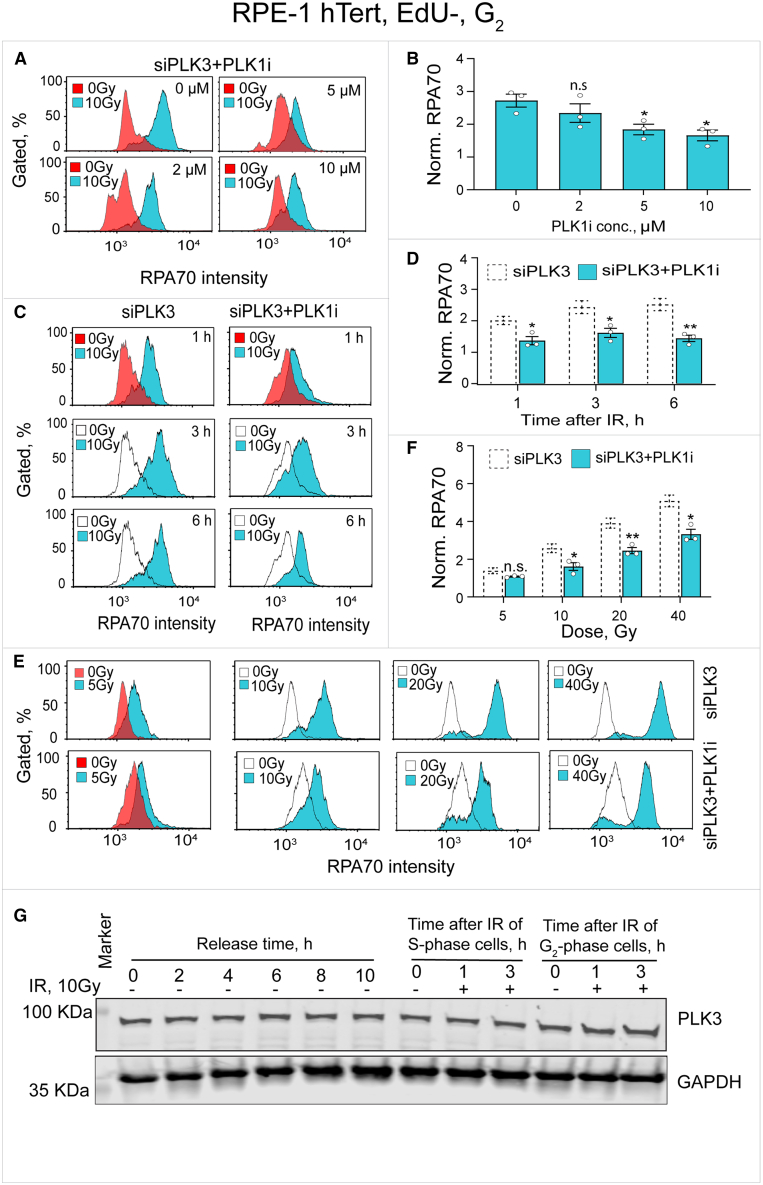
Combined inactivation of PLK1 and PLK3 suppresses resection in EdU− G_2_ phase cells RPE-1 hTert cells were transfected with PLK3-targeting siRNA and subsequently treated with the PLK1i in EdU− G_2_-phase cells. (A) Effect of increasing concentrations of PLK1i on RPA70 intensity in PLK3-depleted cells. (B) Quantification of RPA70 intensity corresponding to (A). (C) Time course analysis of RPA70 accumulation at 1, 3, and 6 h post-10 Gy IR in PLK3-depleted cells, in the presence or absence of 5 μM PLK1i. (D) Quantification of RPA70 intensity corresponding to (C). (E) RPA70 intensity across increasing IR doses (0, 5, 10, 20, and 40 Gy) in PLK3-depleted cells treated with or without 5 μM PLK1i. (F) Quantification of RPA70 intensity corresponding to (E). (G) Western blot analysis of cells released from STB synchronization to examine PLK3 expression level across the cell cycle and after IR exposure. Lane 1 shows the molecular weight marker, lanes 2–7 show PLK3 expression across the S- and G_2_-phase of the cell cycle without IR exposure, and lanes 8–13 show PLK3 expression in cells collected 1 and 3 h post-10 Gy IR after release for 3 or 6 h (representing S and G_2_ phases). (A–F) were performed simultaneously with Figure 4, using the same experimental setup and shared controls. (A, C, and E) show representative flow cytometry plots from an independent biological replicate of the three experiments. Around 10,000 cells were analyzed per sample in each experiment. (B, D, and F) show mean ± SEM from *n* = 3 independent biological replicates. (G) shows representative western blots from three independent experiments. Statistical analyses were performed using an unpaired two-tailed Student’s *t* test. Significance is indicated as follows: ∗*p* < 0.05, ∗∗*p* < 0.01; n.s., not significant.

To confirm this model, PLK1 was inhibited with GSK461364 in PLK3-depleted RPE-1 hTert cells. PLK1i at 5–10 μM effectively suppressed resection in EdU− G_2_-phase cells (Figures 5A and 5B), with inhibition apparent across all test time points post-IR (Figures 5C and 5D) and at IR doses of 5–40 Gy (Figures 5E and 5F). The similarity of resection-suppression by PLK1i in PLK3-depleted cells to PLK1/3i confirms that either kinase can promote resection in G_2_-phase-irradiated cells.

These results confirm that PLK1 inhibition suppresses resection in S-phase-irradiated cells. However, combined PLK1 and PLK3 inhibition is necessary to suppress resection in G_2_-phase-irradiated cells.

We next investigated why PLK3 fails to contribute to resection in S-phase-irradiated cells. PLK3 is reported to be expressed in a cell-cycle-dependent manner.^31^ We speculated therefore that the level of PLK3 might be lower in S-phase than in G_2_-phase. To test this, PLK3 level in RPE-1 hTert cells was monitored after release from STB. Figure S6A shows the cell-cycle progression after release from STB, demonstrating the efficacy of STB in synchronizing the cell cycle, and Figure 5G (lane 2–7) shows that the PLK3 levels are similar in S- and G_2_-phases, ruling out differential expression as the reason for PLK3’s lack of contribution to resection during S-phase irradiation. Another possible explanation is IR-dependent regulation of PLK3 level in EdU+ G_2_-phase cells. To test this, we exposed cells released for 3 and 6 h after STB treatment—corresponding to S and G_2_ phases, respectively—to 10 Gy IR and collected samples at 1 and 3 h post-IR to assess PLK3 levels. Figure S6B confirms successful synchronization, and Figure 5G (lane 8–13) shows that IR fails to alter PLK3 stability/levels in either phase. Collectively, these results indicate that neither cell-cycle–dependent fluctuations nor IR-induced changes in PLK3 levels explain PLK3’s lack of contribution to resection in S-phase-irradiated cells. We conclude that PLK3 is mechanistically excluded from regulating resection in S-phase-irradiated cells by mechanisms that remain to be elucidated.

### Distinct roles of PLK1/3 and the SCF^SKP2^-APC/C^CDH1^ axis in resection regulation in G_2_-phase-irradiated cells

**Figure 6:**
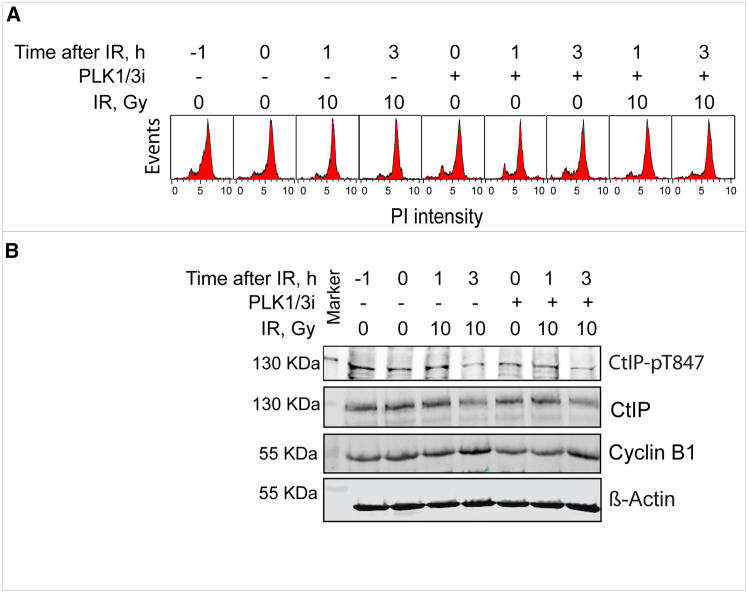
PLK1 and PLK3 regulate resection in G_2_-phase-irradiated cells via a mechanism independent of CtIP degradation (A) Cell-cycle distribution of RPE-1 hTert cells released from STB synchronization and collected at indicated time points. Cells were treated with either PLK1/3 inhibitor (PLK1/3i) at 5 h post-release, exposed to 10 Gy of IR at 6 h post-release, both, or left untreated. (B) Western blot analysis of CtIP, SKP2, and cyclin B1 protein levels in G_2_-phase-enriched cells at 1 and 3 h post-IR exposure, with or without PLK1/3i. Both panels are representative of *n* = 3 independent biological replicates. For flow cytometry analyses, approximately 10,000 cells were analyzed per sample.

PLK1 and PLK3 redundantly regulate resection in G_2_-phase-irradiated cells, but whether they act via the same mechanism as SKP2 depletion, as proposed in the Introduction, remains unclear. Since SKP2 depletion activates the APC/C^CDH1^, causing CtIP destabilization,^17^ we examined CtIP levels in PLK1/3-inhibited, G_2_-phase-enriched RPE-1 hTert cells. Cells were released from STB and 5 h later treated with PLK1/3i. One hour after the addition of the inhibitor, cells were irradiated with 10 Gy and collected at 1 and 3 h to examine CtIP levels. Figure 6A shows that PLK1/3 inhibition did not alter cell-cycle distribution. Figure 6B shows that CtIP levels decreased 3 h after IR in untreated cells, reflecting the activation of the APC/C^CDH1^ following IR.^40^ However, PLK1/3 inhibition does not reduce CtIP levels in unirradiated cells and fails to alter the degradation induced by IR (Figure 6B). In line with this, PLK1/3i fails to modulate the level of cyclin B1—a well-known APC/C^CDH1^ substrate (Figure 6B). Given that PLK3 promotes CtIP-pT847 phosphorylation to activate resection in G_1_-phase cells,^15^ we next assessed CtIP-pT847 in G_2_-phase-irradiated cells. Consistent with total CtIP levels, PLK1/3 inhibition did not affect the responses of CtIP-pT847. Together, these results indicate that PLK1 and PLK3 regulate resection in G_2_-phase cells through a mechanism distinct from the SCF^SKP2^-APC/C^CDH1^-axis-mediated CtIP level regulation.

### PLK1 and PLK3 activity is required for efficient HR and G_2_-phase cell survival

**Figure 7:**
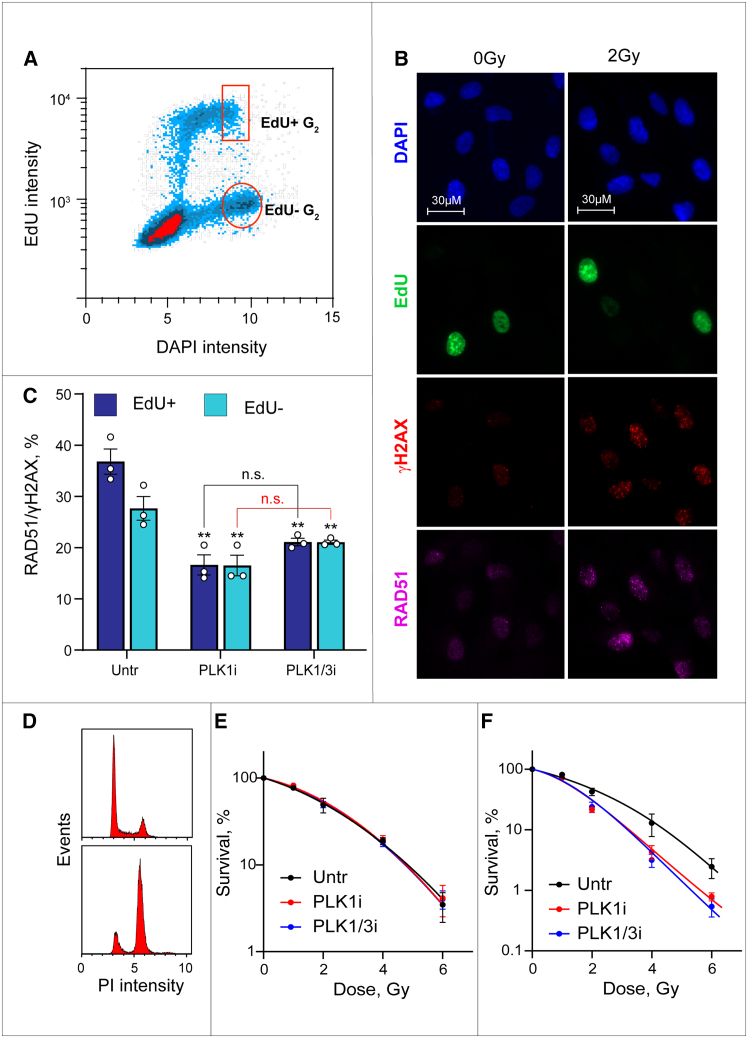
Requirement of PLK1 and PLK3 activity for HR-mediated DSB repair and G_2_-phase cell survival in RPE-1 hTert cells (A) Schematic showing discrimination of EdU-labeled S-phase (EdU+) and G_2_-phase (EdU−)-irradiated cells used for γH2AX and RAD51foci analysis. (B) Representative IF images of γH2AX and RAD51 foci induced by 2Gy IR, treated with PLK1i, PLK1/3i, or left untreated. (C) Quantification of RAD51/γH2AX foci ratio in EdU+ and EdU− G_2_-phase cell. RAD51 and γH2AX foci were measured at 1, 3, and 6 h post-2 Gy IR, and the maximum induction of each marker was used to calculate the ratio (γH2AX maximum typically at 1 h; RAD51 maximum usually at 3 h). The ratio reflects the fraction of DSBs repaired by HR. (D) Flow-cytometry-based cell-cycle distribution of RPE-1 hTert cells, either asynchronously growing or G_2_-phase-enriched, used to confirm cell-cycle status prior to survival analysis in (E and F). (E) Clonogenic survival curve of asynchronously growing cells treated with PLK1i or PLK1/3i and irradiated with indicated doses. Cells were plated 9 h post-IR to allow the repair of DSBs in the presence of inhibitors. (F) Clonogenic survival curve of G_2_-phase-enriched cells treated with PLK1i or PLK1/3i and irradiated with indicated doses. IR to allow the repair of DSBs in the presence of inhibitors. (A, B, and D) show representative data from three independent biological experiments. Approximately 10,000 cells were analyzed per sample in flow cytometry experiments. (C, E, and F) show mean ± SEM from *n* = 3 independent biological replicates. Statistical analyses were performed using an unpaired two-tailed Student’s *t* test. Significance is indicated as follows: ∗*p* < 0.05, ∗∗*p* < 0.01; n.s., not significant.

The RAD51/γH2AX foci ratio is an indicator of HR efficiency, reflecting the fraction of DSBs repaired by HR.^4^^,^^41^^,^^42^ To assess the functional consequences of PLK1 and PLK3 inhibition, RPE-1 hTert cells were pulse-labeled with EdU prior to 2 Gy IR to distinguish S-phase-derived (EdU+) and non-S-phase (EdU−) populations, using a protocol analogous to the resection assay (Figure 7A). RAD51 and γH2AX foci were simultaneously measured (Figure 7B) at 1, 3, and 6 h post-IR, and the maximum induction levels of each marker were used to calculate the RAD51/γH2AX ratio (γH2AX maximum typically at 1 h; RAD51 maximum usually at 3 h).

PLK1/3 inhibition (PLK1/3i) consistently reduced the RAD51/γH2AX ratio in both EdU+ and EdU− G_2_-phase cells, indicating impaired HR. PLK1 inhibition alone also decreased the ratio significantly, including in EdU− G_2_-phase cells where resection is only modestly affected (Figure 7C), suggesting that PLK1 promotes HR through mechanisms beyond resection, consistent with its role in facilitating phosphorylation of RAD51 at Ser14 to promote full activation.^43^^,^^44^

Clonogenic survival assays mirrored these findings. In asynchronously growing cells (confirmed by flow cytometry; Figure 7D), neither PLK1i nor PLK1/3i markedly affected the radiosensitivity (Figure 7E). In contrast, in G_2_-phase-enriched populations (confirmed by flow cytometry; Figure 7D), both inhibitors sensitized cells to IR in a similar degree (Figure 7G). These findings reveal that PLK1 and PLK3 support HR-mediated DSB repair and G_2_-phase survival.

## Discussion

### Complementary regulation by PLK1 and PLK3 of DNA damage signaling

The PLK family comprises evolutionarily conserved serine/threonine kinases coordinating key processes in cell-cycle progression and the DDR. Among the five mammalian isoforms (PLK1–PLK5), PLK1 and PLK3 are the most extensively characterized, performing distinct yet complementary tasks.

PLK1 is a prototypical mitotic kinase with cell-cycle-dependent expression.^45^ Its levels are minimal in G_1_, rise during S, and peak in G_2_ and mitosis, where it orchestrates centrosome maturation, Wee1-like protein kinase (WEE1) inhibition, cell-division cycle 25C (Cdc25C)-CDK1 activation, and essential mitotic processes including spindle assembly, chromosome segregation, and cytokinesis.^46^ Following mitosis, PLK1 is degraded via APC/C^CDH1^, ensuring proper mitotic exit.^47^ PLK3, structurally similar to PLK1, exhibits distinct regulatory characteristics. Earlier studies suggested PLK3 peaks in G_1_ and early S phase, but our analyses and recent reports indicate that PLK3 protein levels remain largely constant from S through G_2_.^48^ PLK3 facilitates S-phase entry by upregulating cyclin E1 and activating CDK2 via Cdc25A^31^ and promotes mitotic entry through Cdc25C phosphorylation.^49^ PLK3 also contributes to spindle assembly, although overexpression can induce mitotic errors and apoptosis.^50^

Both PLK1 and PLK3 have also been implicated in DDR, where they act oppositely. PLK1 is normally activated via Thr210 phosphorylation to promote mitosis,^51^ but DNA damage rapidly suppresses this modification in an ataxia telangiectasia Mutated (ATM)-dependent manner, facilitating cell-cycle arrest.^52^^,^^53^ On the other hand, PLK1 restrains ATM signaling; thus, its inhibition in turn further enhances ATM and checkpoint kinase 2 (CHK2) phosphorylation following irradiation and strengthens cell-cycle arrest.^54^ Additionally, in G_2_-phase cells, DSBs trigger PLK1 degradation through APC/C^CDH1^, promoting ATM and Rad3-related (ATR)-mediated checkpoint kinase 1 (CHK1) phosphorylation, consolidating the G_2_/M checkpoint.^20^ Together, these mechanisms form a feedback loop wherein ATM-dependent PLK1 downregulation amplifies DDR signaling and checkpoint enforcement. PLK3, in contrast, is activated in response to IR in an ATM-dependent manner.^55^ Active PLK3 phosphorylates CHK2 at Ser62 and Ser73, priming PLK3 for full ATM-mediated activation.^56^ This cascade phosphorylates CDC25C and enforces the G_2_/M checkpoint. Unlike PLK1, whose activity is suppressed by DNA damage, PLK3 activation provides a compensatory mechanism in G_2_-phase cells, ensuring proper checkpoint and DNA repair function.

In this study, we demonstrate that PLK1 and PLK3 regulate resection redundantly in G_2_-phase-irradiated cells, whereas PLK3 is not required in S-phase-irradiated cells. This difference cannot be explained by changes in protein levels, as PLK3 abundance is comparable between S- and G_2_-phase cells even in the presence of IR (Figure 5G). Cell-cycle-specific activation alone is also unlikely to account for our findings: PLK3 should have already been activated in S-phase cells since PLK3 functions as promoter for both S-phase and mitotic entry.^31^^,^^49^ Instead, the distinct contributions of PLK1 and PLK3 may reflect differences in DDR signaling between cell-cycle phases. In S-phase-irradiated cells, the DDR is predominantly ATR-dependent, with a limited contribution from ATM.^57^ In contrast, G_2_-phase cells rely on coordinated ATM and ATR signaling.^37^ Given that PLK3 activation is ATM-dependent, its contribution to resection may be limited in S-phase-irradiated cells, whereas in G_2_-phase-irradiated cells, stronger ATM engagement may enable PLK3 to function redundantly with PLK1.

### PLK1 and PLK3 regulate resection through a pathway distinct from APC/C^CDH1^

While SKP2 loss in G_2_-phase cells suppresses resection by activating the APC/C^CDH1^ and promoting CtIP degradation, combined PLK1 and PLK3 inhibition also impairs resection without altering CtIP stability, indicating a mechanism distinct from the SCF^SKP2^-APC/C^CDH1^ pathway. One possibility is that PLK1 and PLK3 directly phosphorylate key resection factors or associated proteins to modulate their activity or recruitment. CtIP is one such candidate, but our analysis showed that PLK1/3 inhibition does not affect CtIP phosphorylation at T847 before and after IR, suggesting that its involvement may be via other phosphorylation sites or protein interaction mechanisms. The potential contribution of other regulators, such as BRCA1, MRE11, and EXO1, remains to be explored.

Furthermore, the interplay between PLK1/PLK3 and the ATM–CHK2/ATR–CHK1 pathways suggests that PLK-mediated signaling integrates with broader DDR networks coordinating IR-induced G_2_/M phase checkpoint control and resection. We recently reported a strong positive correlation between resection and G_2_/M checkpoint activation following IR, emphasizing their functional coupling.^37^ Therefore, PLK1 and PLK3 may influence resection indirectly by regulating these key checkpoint kinases rather than by directly modulating resection factors. Additionally, this regulatory axis positions PLK1 and PLK3 as critical mediators linking resection with G_2_ checkpoint enforcement. Understanding this interplay could provide important mechanistic insights into how cells coordinate DNA repair with cell-cycle progression and identifies potential targets for further study.

### Biological consequence of PLK1 and PLK1/3 inhibition in G_2_ phase cells

While our data show that both PLK1 and PLK1/3 inhibition impairs HR efficiency and G_2_-phase cell survival, they do not fully reveal the biological consequences of resection downregulation. Notably, PLK1i significantly reduces the RAD51/γH2AX ratio in G_2_-phase-irradiated cells to a similar extent as PLK1/3i, despite minimal effects on resection. This suggests that the additional resection defect induced by PLK1/3i does not further suppress HR. Consistently, PLK1i sensitizes G_2_-phase cells to IR to a similar degree as PLK1/3i, indicating that resection downregulation contributes little additional effect on radiosensitivity. Together, these observations suggest that the effects of PLKs inhibition on HR and survival are largely independent of resection regulation.

Resection is a critical determinant of DSB repair pathway choice, promoting not only HR but also SSA and alt-EJ. SSA, in particular, requires more extensive resection to expose long 3′ ssDNA regions for annealing.^58^^,^^59^ RAD51 loading on resected ssDNA facilitates HR while simultaneously suppressing SSA and alt-EJ, highlighting the competitive nature of these repair pathways.^60^^,^^61^ Based on this, we propose that PLK-dependent regulation of resection may influence the balance among DSB repair pathways. Under conditions of partial resection impairment caused by PLK1/3i, HR may already be compromised, while SSA—being more resection-dependent—could be even more strongly affected. This suggests that PLK1/3 inhibition may suppress multiple resection-dependent repair pathways, especially SSA. Conversely, PLK1i leaves resection largely intact but compromises HR by affecting RAD51 accumulation, which could potentially upregulate SSA. Although SSA was not directly measured in this study, these considerations provide a mechanistic explanation for the biological consequences of impaired resection caused by PLKs inhibition and highlight an important area for future investigation.

### Cell-cycle-specific regulation of resection in S- and G_2_-phase-irradiated cells

Resection generates a 3′ ssDNA overhang, suppressing NHEJ and promoting homology-directed DSB repair, which is tightly cell-cycle regulated: most active during G_2_, limited activity in G_1_, and nearly absent in G_0_.^14^^,^^15^^,^^32^ A central mechanism enforcing this phase specificity is CDK-mediated control of resection factors. CDKs regulate resection by phosphorylating multiple targets. One key effector is CtIP, whose phosphorylation at Thr847 and Ser327 is essential for resection initiation.^62^^,^^63^^,^^64^ CDK2 also phosphorylates CtIP at Ser276 and Thr315, promoting its interaction with PIN1 and paradoxically limiting resection.^65^ Beyond CtIP, CDKs also regulate nucleases and cofactors including EXO1,^66^ DNA2,^67^ and NBS1.^68^ Protein stability adds a second layer of control: APC/C^CDH1^ mediates the degradation of CtIP in G_1_/G_0_ cells, which effectively suppresses resection.^32^^,^^40^ In contrast, the SCF^SKP2^ inhibits APC/C^CDH1^ activity during G_2_, stabilizing CtIP to support HR-mediated repair.^17^ EXO1 is similarly regulated by E3 ligase pathways.^69^^,^^70^^,^^71^

While these mechanisms establish the general framework of cell-cycle-dependent resection, how resection is coordinated depending on the cell-cycle phase at the time of irradiation—S versus G_2_—remained unclear. Our previous study addresses this by analyzing resection in a cell-cycle-phase-specific manner, precisely tracking both the timing of irradiation and where resection occurs. In G_2_-phase cells exposed to low-dose IR, ATM and ATR cooperate epistatically to regulate resection, whereas at higher doses, they start acting independently.^37^ Notably, in S-phase-irradiated cells, resection occurs without ATR-activation requirement, and inhibition of ATM increase, rather than decrease resection level.^57^ This study provides the first clear evidence that distinct, phase-specific mechanisms govern resection in S- and G_2_-phase-irradiated cells.

A unique and specific mechanism regulating resection was uncovered in our subsequent work, which operates exclusively in G_2_-phase-irradiated cells.^17^ Disruption of SCF^SKP2^ by SKP2 depletion abrogates resection in G_2_-phase-irradiated cells by facilitating CtIP degradation through APC/C^CDH1^. Notably, this requirement is strictly limited to cells irradiated during the G_2_-phase, whereas cells irradiated in S-phase still undergo efficient resection upon entering G_2_, independently of SCF^SKP2^ activity. The G_2_-phase specificity in the regulation of resection by SCF^SKP2^ remains unclear, but it can be rationalized by the observation that resection in S/G_2_ relies on the transient (1–3 h) stabilization of CtIP, protecting it from APC/C^CDH1^-mediated degradation. This stabilization might be redundant in S-phase, where APC/C^CDH1^ activity is constitutively low, thus bypassing the need for SCF^SKP2^-mediated stabilization. Indeed, activated CHK1 in S-phase was found to phosphorylate CDH1, promoting its degradation by SCF^β−TRCP^.^72^

Our current study further uncovers a distinct, cell-cycle-specific role for PLK1 and PLK3 in regulating resection. In S-phase-irradiated cells, PLK1 activity is essential for efficient resection across radiation doses, indicating an important and non-redundant role for PLK1 during S-phase. In contrast, PLK1 inhibition alone does not affect resection in G_2_-phase-irradiated cells. Instead, impairing resection in G_2_-phase-irradiated cells requires the combined inactivation of both PLK1 and PLK3, revealing a functional redundancy or compensatory mechanism between the two kinases during this phase. Importantly, this G_2_-phase-specific requirement for PLK3 cannot be explained by changes in protein abundance, suggesting regulation at the level of kinase activity or substrate interaction. These findings demonstrate that PLK1 and PLK3 contribute to resection through distinct, phase-dependent mechanisms—PLK1 being dominant in S-phase and sharing function with PLK3 in G_2_-phase.

### Limitations of the study

While our study clarifies the functional roles of PLK1 and PLK3 in resection, several limitations remain. First, although we identify a distinct cell-cycle-dependent requirement—where PLK1 is essential in S-phase but functionally redundant with PLK3 in G_2_-phase—the molecular “switch” governing this transition remains to be characterized. Our data suggest that this is not driven by changes in protein abundance, pointing instead to phase-specific kinase activation or differential substrate accessibility that has yet to be mapped.

Second, while we demonstrate that PLK1/3 inhibition suppresses resection independently of the SCF^SKP2^-APC/C^CDH1^ axis, the downstream targets mediating this regulatory pathway remain undefined. It is currently unclear whether PLK1 and PLK3 directly phosphorylate CtIP to modulate its activity or act through other resection factors such as DNA2 or EXO1.

Finally, although pharmacological inhibitors provided the temporal resolution necessary to interrogate S- and G_2_-phase-specific functions, complementary genetic approaches—such as the analysis of non-phosphorylatable mutants of candidate PLK1/3 substrates—would further refine our understanding of how these kinases coordinate resection across the cell cycle.

## Resource availability

### Lead contact

Further information and requests for resources and reagents should be directed to and will be fulfilled by the lead contact, Fanghua Li (fanghua.li@uk-essen.de).

### Materials availability

This study did not generate new unique reagents.

### Data and code availability

• The data reported in this paper will be shared by the lead contact upon request.
• This paper does not report original code.
• Any additional information required to reanalyze the data reported in this paper is available from the lead contact upon request.

## Acknowledgments

This work was supported by grants from the 10.13039/501100002347BMBF (02S8254, 02S8467, 03NUK005C, and 02NUK043B), 10.13039/501100001659DFG GRK1739, and the 10.13039/501100008436Ernst und Berta Grimmke-Stiftung (grants Lfd19/22 and Lfd. 19/24).

## Author contributions

B.P., conceptualization, formal analysis, investigation, methodology, and visualization; F.L., conceptualization, formal analysis, investigation, methodology, visualization, writing – review and editing, supervision, and funding acquisition; E.M., formal analysis, investigation, methodology, and visualization. M.S., project administration, supervision, and funding acquisition; B.T., project administration, supervision, and funding acquisition; G.I., conceptualization, writing – review and editing, supervision, project administration, and funding acquisition.

## Declaration of interests

The authors declare no competing interests.

## STAR★Methods

### Key resources table

**Table:** 

REAGENT or RESOURCE	SOURCE	IDENTIFIER
**Antibodies**		

Mouse monoclonal anti-RPA70B	Dr. J. Hurwitz^73^	N/A
Mouse monoclonal anti-BrdU	BD Biosciences	Cat# 347580; RRID: AB_400326
Mouse monoclonal anti-CTIP	Santa Crutz Biotechnology	Cat# sc-271339; RRID: AB_10608728
Mouse monoclonal anti-RAD51	GeneTex	Cat # GTX70230; RRID: AB_372856
Rabbit polyclonal anti- γH2AX	GeneTex	Cat # GTX127342;RRID: AB_2833105
Rabbit monoclonal anti-PLK3	Cell Signaling Technology	Cat# 4896; RRID: AB_10544409
Mouse monoclonal anti-CCNB1	Santa Cruz Biotechnology	Cat# sc-245; RRID: AB_627338
Mouse monoclonal anti-GAPDH	UBP Bio	Cat# Y1041; RRID: AB_3086807
Rabbit polyclonal anti-ß-Actin	Proteintech	Cat# 20536-1-AP; RRID: AB_10700003
Rabbit polyclonal anti-CtIP-pT847	Custom made by Genscript	This study
Alexa Fluor 488 Goat anti-Mouse IgG (H+L)	Thermo Fisher Scientific	Cat# A 11001; RRID: AB_2534069
Alexa Fluor 647 Goat anti-Mouse IgG (H+L)	Thermo Fisher Scientific	Cat# A 21245; RRID: AB_2535813
Alexa Fluor 555 Goat anti-Mouse IgG (H+L)	Thermo Fisher Scientific	Cat# A 21428; RRID: AB_2535849
IRDye 680RD Goat anti-Mouse IgG (H+L)	LI-COR Biosciences	Cat#926-68020; RRID: AB_10706161
IRDye 800CW Goat anti-Mouse IgG (H+L)	LI-COR Biosciences	Cat#926-32210; RRID: AB_621842
IRDye 680RD Goat anti-Rabbit IgG (H+L)	LI-COR Biosciences	Cat#926-68021; RRID: AB_10706309
IRDye 800CW Goat anti-Rabbit IgG (H+L)	LI-COR Biosciences	Cat#926-32211; RRID: AB_621843

**Chemicals, peptides, and recombinant proteins**		

BrdU	Sigma-Aldrich	Cat#B5002
EdU	Thermo Fisher Scientific	Cat# A10044
PI	Thermo Fisher Scientific	Cat# P1304MP
Non-Essential Amino Acid	Merke	Cat#K0293
Thymidine	Sigma-Aldrich	Cat# T1895
Triton X-100	ROTH	Cat#3501.4
GSK461364	SelleckChem	Cat#S2193
GW 843682X	Tocris Bioscience	Cat#2977
RIPA buffer	Thermo Fisher Scientific	Cat#89900
BSA Fraction V	PAN Biotech	Cat#P06-1391015
Cold-water fish skin gelatin	Merke	Cat#G7041
Sucrose	ROTH	Cat#9097.1
Paraformaldehyde	ROTH	Cat#0335.3

**Critical commercial assays**		

Click-IT EdU Alexa Fluor 647 Flow Cytometry Assay Kit	Thermo Fisher Scientific	Cat# C10340
Click-IT EdU Alexa Fluor 488 Flow Cytometry Assay Kit	Thermo Fisher Scientific	Cat# C10337

**Experimental models: Cell lines**		

RPE-1 hTert	ATCC	ATCC CRL-4000
82-6 hTert	Markus Löbrich^74^	N/A

**Oligonucleotides**		

siRNA targeting PLK3: CUGCAUCAAGCAGGUUCACUA	Barton et al.^30^	N/A
negative control siRNA: UUCUCCGAACGUGUCACGU	Forment et al.^75^	N/A

**Software and algorithms**		

Kaluza	Beckmann Coulter	RRID:SCR_016182
Imaris	Bitplane	RRID: SCR_007370
GraphPad Prism	GraphPad Software, LLC	RRID: SCR_002798

**Others**		

Flow cytometer	Beckmann Coulter	Cat#B43618
Nucleofector	Lonza	Cat#AAB-1001
Odyssey infrared scanner	Li-COR Biosciences	https://www.licor.com/bio/odyssey-clx/

### Experimental model and study participant details

Cells were cultured at 37°C in a humidified atmosphere containing 5% CO_2_. Human retinal pigment epithelial RPE-1 hTERT cells (ATCC CRL-4000; female) were cultured in Dulbecco’s Modified Eagle’s Medium (DMEM) supplemented with 10% fetal bovine serum (FBS). 82-6 hTert (human fibroblasts, male; kindly provided by Drs. Löbrich and Jeggo^73^) were maintained in Minimum Essential Medium (MEM) supplemented with 10% fetal bovine serum (FBS) and 1% non-essential amino acids. RPE-1 hTert were authenticated by ATCC through short tandem repeat (STR) profiling. Both cell lines were routinely tested for mycoplasma contamination, and only mycoplasma-free cultures were used for experiments.

#### Irradiation

Cells were irradiated at room temperature (RT) using a 320 kV X-ray machine operated with a 1.65 mm aluminum filter (GE Healthcare). The dose rate was 3.2 Gy/min at a distance of 500 mm from the source, and 1.4 Gy/min at 750 mm. A rotating irradiation table ensured uniform dose distribution within a defined ring-shaped exposure field.

#### RNA interference

To deplete relevant target proteins, knockdown experiments were performed using the following specific siRNAs: Negative control (NC) (UUCUCCGAACGUGUCACGU),^74^ PLK3 (CUGCAUCAAGCAGGUUCACUA).^30^ The siRNAs were delivered by nucleofection using the Nucleofector-2B device (Lonza). The program X-020 was used for RPE-1 hTert cells, and T-030 for 82-6-hTert. The knockdown efficiency was assessed through quantitating protein levels by western blotting (WB) 48 hours (h) after nucleofection.

#### Treatment of cells with kinase inhibitors

(R)-5-(6-((4-methylpiperazin-1-yl) methyl)-1H-benzo[d]imidazol-1-yl)-3-(1 (2(trifluoromethyl) phenyl) ethoxy) thio- phene -2-carboxamide (GSK461364, PLK1 inhibitor, to be termed here PLK1i, SelleckChem; IC50 PLK1=2.2nM, at least 390-fold greater selectivity for PLK1 than for PLK2 and PLK3) was dissolved in DMSO (Sigma-Aldrich) at 10 mM. 5-(5,6-Dimethoxy-1H-benzimidazol-1-yl)-3-[[2-(trifluoromethyl) phenyl] methoxy]-2 thiophe- necarbox- carboxamide (GW 843682X, PLK1/3, to be termed here PLK1/3i, Tocris Biotechnique; IC_50_ PLK1=2.2nM; IC_50_ PLK3=9.1nM) was dissolved in DMSO (Sigma-Aldrich) at 10 mM.

#### Flow cytometry (FC) analysis of resection

Resection was assessed via RPA70 detection and 5-bromo-2′-deoxyuridine (BrdU) ssDNA exposure assays. In both assays, exponentially growing cells were pulse-labeled with 5 μM 5-ethynyl-2′-deoxyuridine (EdU) for 30 min. After labeling, the medium was removed, and cells were rinsed once with pre-warmed PBS. Fresh complete growth medium was added, and cells were irradiated with X-rays. For BrdU-based detection of ssDNA, cells were incubated with 10 μM BrdU for 24 h prior to EdU labeling. At the indicated time points post-IR, cells were harvested by trypsinization. Cell pellets were incubated for 4 min in ice-cold PBS containing 0.2% Triton X-100, consistent with pre-extraction approaches used in previous protocols^75^ but at a concentration optimized for our cell system. Following centrifugation (5 min), the pellets were fixed in 3% paraformaldehyde (PFA) supplemented with 2% sucrose in PBS for 15 min. Cells were then blocked overnight at 4°C in PBG buffer (0.2% cold-water fish skin gelatin and 0.5% BSA fraction V in PBS). Cells were incubated for 1.5 h with a monoclonal antibody against RPA70,^76^ or BrdU (BD biosciences, San Jose, CA, USA, 347580). washed twice with PBS, and subsequently incubated for 1.0 h with an Alexa Fluor 488-conjugated secondary antibody (Thermo Fisher Scientific, Waltham, Massachusetts, USA, A-11001). EdU signals were developed using an EdU staining kit (Thermo Fisher Scientific, C10340), following the manufacturer’s instructions. Finally, cells were stained with 40 μg/ml propidium iodide (PI, Sigma-Aldrich) for 15 min at RT. Three-parameter analysis was performed using a Gallios flow cytometer (Beckman Coulter, Brea, CA, USA). EdU-negative G_1_ and G_2_ cells were distinguished based on PI fluorescence intensity. Data were analyzed using Kaluza 2.1 software (Beckman Coulter). At least 10,000 cells were analyzed per sample, and all RPA70 and BrdU measurements were normalized to the arithmetic mean of the 0 Gy (unirradiated) control within each experiment and are presented as fold-change relative to this control.

#### RAD51 and γH2AX foci determination by immunofluorescence (IF)

RPE-1 hTert cells were grown on poly-L-lysine–coated coverslips. Thirty minutes prior to irradiation, cells were pulse-labeled with 2 μM EdU to mark S-phase populations. Cells were then irradiated with 2 Gy X-rays, and EdU was washed out by replacing the medium with fresh EdU-free growth medium. Cells were next fixed in 3% paraformaldehyde (PFA) with 2% sucrose in PBS for 15 min at RT. Fixed cells were permeabilized for 10 min in P-Solution (50 mM EDTA, pH 8.0; 50 mM Tris-HCl, pH 7.6) containing 0.5% Triton X-100, and subsequently blocked overnight at 4°C in PBG buffer (0.2% fish-skin gelatin, 0.5% BSA in PBS). For simultaneous detection of RAD51 and γH2AX, cells were incubated with mouse monoclonal anti-RAD51 antibody (GeneTex, Irvine, CA, USA, GTX70230; 1:400) and rabbit polyclonal anti-γH2AX antibody (GeneTex, GTX127342; 1:400) diluted in PBG for 1.5 h at RT. After washing three times with PBS, cells were incubated with anti-rabbit Alexa Fluor 555 (Thermo Fisher Scientific, A-21428) and anti-mouse Alexa Fluor 647 (Thermo Fisher Scientific, A-21245) conjugated secondary antibodies for 1.5 h at RT (1:400 in PBG). EdU incorporation was visualized using the Click-iT EdU staining kit (Thermo Fisher Scientific, C10337), and nuclei were counterstained with 0.2 μg/mL DAPI for 10 min at RT. Coverslips were mounted with PromoFluor antifade mounting medium (PromoCell, Heidelberg, Germany). Fluorescence images were acquired using an AxioScan.Z1 (Carl Zeiss, Oberkochen, Germany) scanning areas of 4 mm ×4 mm, containing 10,000–30,000 cells. Quantitative image-based cytometry (QIBC) combining EdU and DAPI signals was used to discriminate cell cycle phases at the time of irradiation. Cellular segmentation was performed using Imaris 9.5.1 software (Bitplane, Zürich, Switzerland), and data were exported and analyzed using Kaluza 2.1 software (Beckman Coulter). Foci numbers were background-corrected by subtracting the corresponding 0 Gy control for each condition.

#### Polyacrylamide gel electrophoresis (SDS-PAGE) and WB

Cells were harvested and washed twice with ice-cold PBS. Approximately 5 × 10^6^ cells were lysed for 30 min in 200 μL of ice-cold RIPA buffer (Thermo Fisher Scientific), supplemented with Halt™ phosphatase (Thermo Fisher Scientific) and protease inhibitor cocktails (Thermo Fisher Scientific), as recommended by the manufacturer. Lysates were centrifuged at 12,000 Revolutions Per Minute for 15 min at 4°C, and protein concentrations in the supernatants were determined using the Bradford assay. Standard protocols were followed for SDS-PAGE and immunoblotting. Unless stated otherwise, 50 μg of total protein from RIPA whole-cell extracts was loaded per lane. Proteins were transferred onto nitrocellulose membranes and incubated with primary and secondary antibodies following standard procedures. The primary antibodies used were: anti-PLK3 (Cell Signaling Technology, Danvers, MA, USA, #4896), anti-CtIP (Santacruz Biotechnology, Dallas, TX, USA, sc-271339), anti-CCNB1 (Santa cruz Biotechnology, sc-245), anti-ß-Actin (Proteintech Group, Rosemont, IL, USA, 20536-1-AP) and anti-GAPDH (UBP Bio, Dallas, TX, USA, Y1041). Secondary antibodies included anti-mouse IgG and anti-rabbit IgG conjugated with IRDye 680 or IRDye 800 (LI-COR Biosciences, Lincoln, NE, USA; #92668020, #92632210, #92668021, #92632211). Immunoblots were visualized using an Odyssey infrared imaging system (LI-COR Biosciences).

#### Clonogenic survival assay

To assess cell survival following DNA damage, RPE-1 hTert cells were treated with the indicated inhibitors and irradiated with X-rays. Nine hours post-IR, cells were plated at appropriate densities in fresh growth medium to allow colony formation. Under this experimental setup, DSB repair occurred in the presence of the inhibitors before plating. Colonies were allowed to form for 10–14 days, stained with crystal violet, and counted. Survival fractions were calculated relative to unirradiated controls.

#### Enrichment of cells in S- and G_2_ phase

To enrich RPE-1 hTert cells in S- and G_2_ phase, cells were subjected to a single thymidine block (STB), as described previously.^17^^,^^40^ Cells were incubated with 2 mM thymidine for 18 h. Afterward, cells were washed twice with pre-warmed PBS and released into fresh growth medium to resume cell cycle progression. Cells were harvested for experiments at 3 h (S-phase enrichment) or 6 h (G_2_-phase enrichment) post-release. Irradiation and subsequent sample collection for western blotting were performed either immediately or at the indicated time points.

### Quantification and statistical analysis

All data are presented as mean ± standard error of the mean (SEM). Each experiment was performed with *n* = 3 independent biological replicates, as indicated in the corresponding figure legends.

Statistical analyses were conducted using GraphPad Prism (version 10.1.2; GraphPad Software, San Diego, CA, USA). Comparisons between two groups were performed using an unpaired two-tailed Student’s *t*-test.

Statistical details, including the exact value of *n*, statistical tests used, and significance levels, are provided in the figure legends. A *p*-value < 0.05 was considered statistically significant. Significance levels are denoted as follows: *p* < 0.05 (∗), *p* < 0.01 (∗∗), and n.s., not significant.

## Supplemental information

Document S1. Figures S1–S6

Data S1. Original uncropped western blot images, related to Figures 4A, 5G, and 6B

Data S2. Original uncropped microscopy images, related to Figure 7B
