# Supplementary material for: Cell-cycle-dependent regulation of DNA end resection by PLK1 and PLK3 without CtIP level modulation
Source: iScience. 2026 Jul 1;29(7):116450. doi: 10.1016/j.isci.2026.116450 (PMC13355021; doi:10.1016/j.isci.2026.116450)
Supplement: Document S1. Figures S1–S6 [file mmc1.pdf]

iScience, Volume *29*

Supplemental information

Cell-cycle-dependent regulation of DNA end resection by PLK1 and PLK3 without CtIP level modulation

Bing Pan, Fanghua Li, Emil Mladenov, Martin Stuschke, Beate Timmermann, and George Iliakis

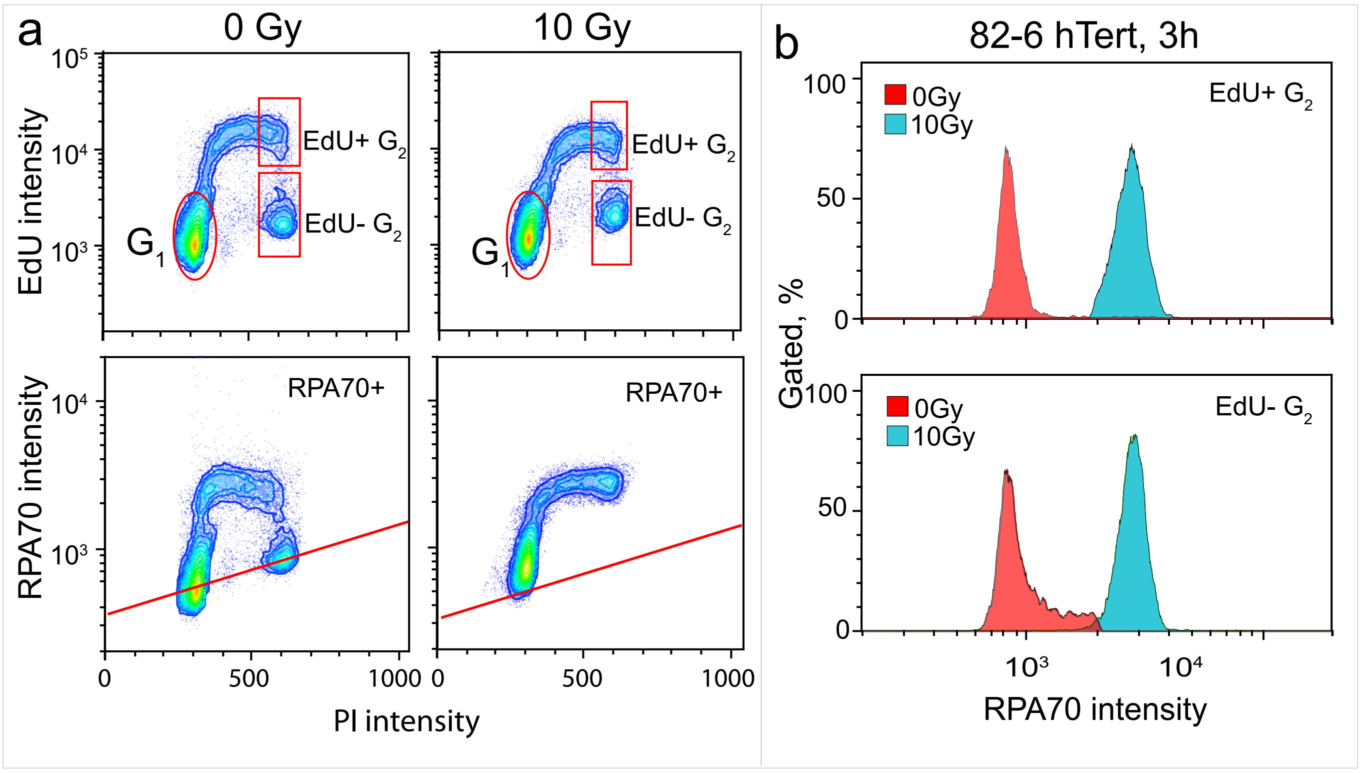

Figure S1. Pan et al.

Figure S1. Gating strategy and validation of RPA70 signal as a marker of resection in G_2-phase cells irradiated during S or G_2 phase. (a) Flow cytometry gating strategy used to identify G_2-phase cells based on PI staining intensity and to distinguish EdU-positive (EdU+) and EdU-negative (EdU-) populations. EdU labeling was performed immediately before irradiation to mark cells in S-phase at the time of IR exposure. (b) Representative flow cytometry histograms showing chromatin-bound RPA70 levels in G_2-phase cells 3 h post-10 Gy IR exposure, compared to unirradiated controls. The robust increase in RPA70 signal validates this method for assessing resection while retaining information on the cell cycle phase during irradiation. Panel (b) is representative of n=3 independent biological replicates. Approximately 10,000 cells were analyzed per sample in each experiment.

82-6 hTert, EdU+, G_2

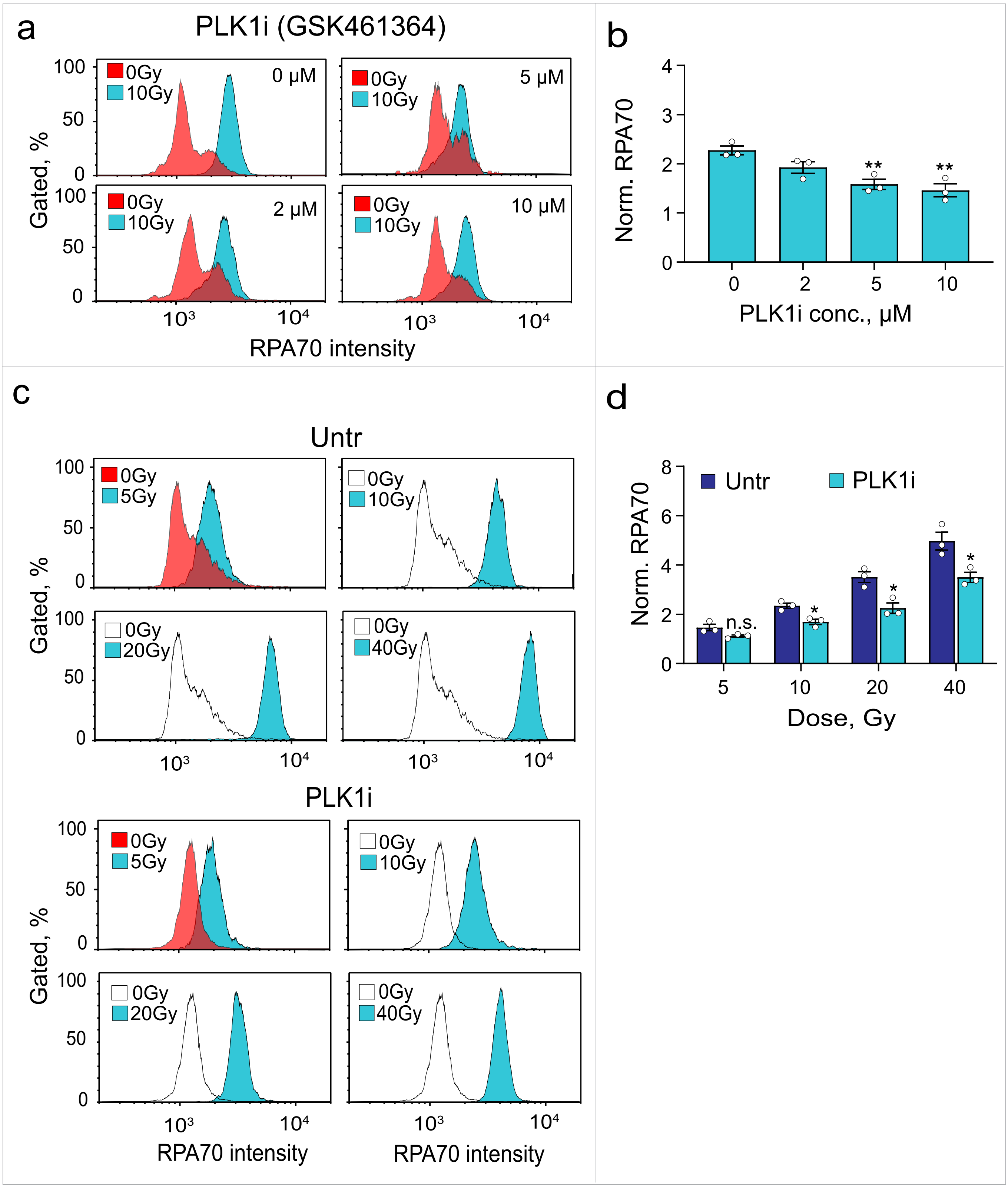

Figure S2, Pan et al.

Figure S2. PLK1 inhibition with GSK461364 (PLK1i) suppresses resection in 82-6 hTert cells irradiated during S-phase and analyzed in G_2-phase. 82-6 hTert cells were pulse-labeled with EdU to mark cells in S-phase at the time of IR exposure and subsequently analyzed during G_2-phase. (a) Representative flow cytometry plots showing RPA70 intensity in cells treated with increasing concentrations of PLK1i (0, 2, 5, or 10 µM) following 10 Gy IR. (b) Quantification of RPA70 intensity corresponding to panel (a). (c) Representative flow cytometry plots showing the effect of 5 µM PLK1i on RPA70 intensity after exposure to different doses of IR (0, 5, 10, 20, and 40 Gy). (d) Quantification of RPA70 intensity corresponding to panel (c). Panels (a) and (c) show representative flow cytometry plots from three independent biological replicates. Approximately 10,000 cells were analyzed per sample in each experiment. Panels (b) and (d) show mean ± SEM from n = 3 independent biological replicates. Statistical analyses were performed using an unpaired two-tailed Student's *t*-test. Significance is indicated as follows: $p < 0.05$ (*), $p < 0.01$ (**), and n.s., not significant.

82-6 hTert, EdU-, G_2

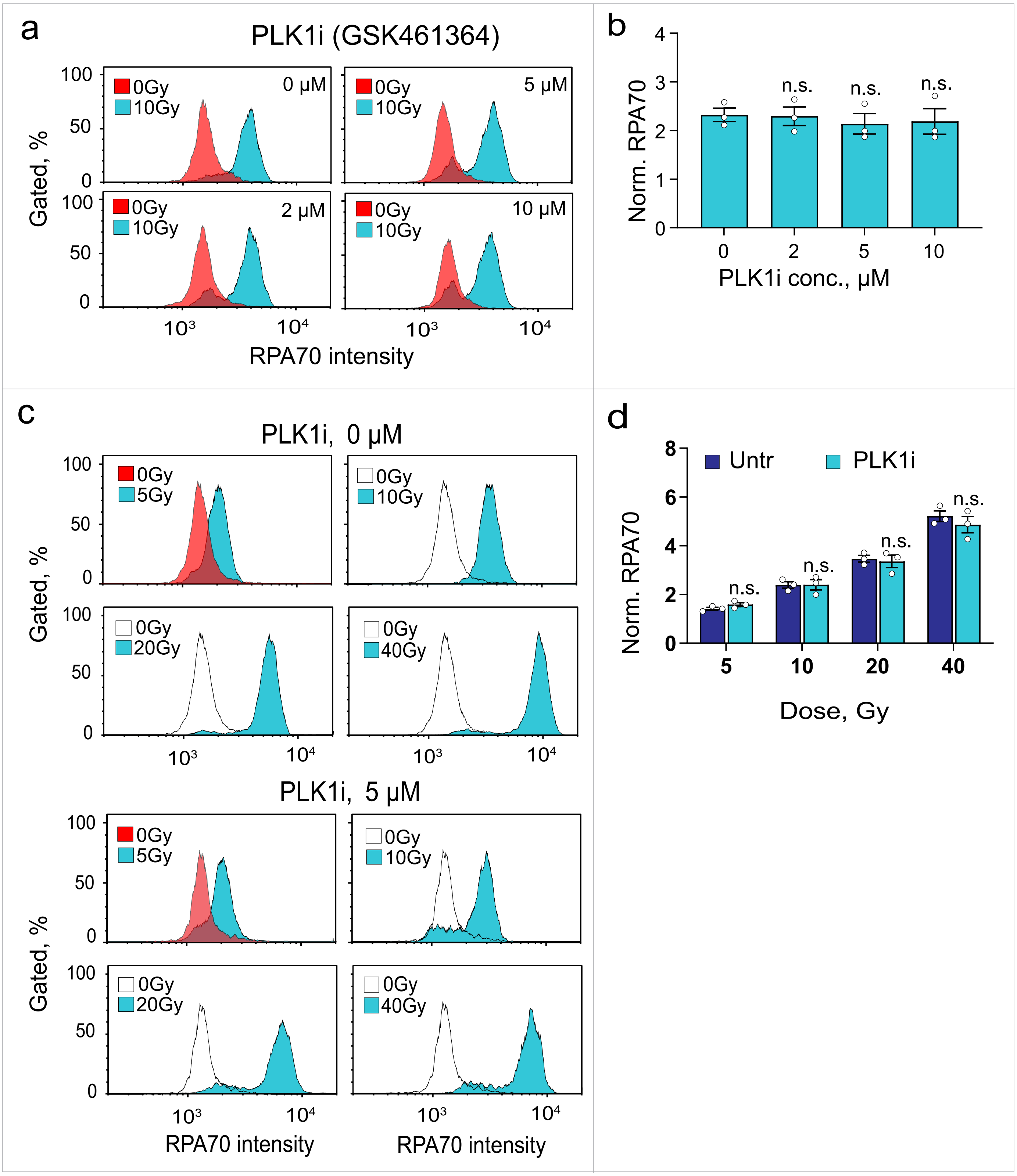

Figure S3, Pan et al.

Figure S3. PLK1i fails to suppress resection in 82-6 hTert cells irradiated during G_2-phase (EdU- G_2 cells). Experiments were performed with the same panel layout as Figure S2. EdU-negative (EdU-) G_2-phase cells, representing cells irradiated during G_2, were analyzed for resection using chromatin-bound RPA70 intensity measured by flow cytometry. (a, c) Representative flow cytometry plots showing RPA70 intensity after treatment with increasing concentrations of PLK1i or different IR doses. (b, d) Quantification of RPA70 intensity corresponding to panels (a) and (c). Panels (a) and (c) show representative flow cytometry plots from three independent biological replicates. Approximately 10,000 cells were analyzed per sample in each experiment. Panels (b) and (d) show mean ± SEM from n = 3 independent biological replicates. Statistical analyses were performed using an unpaired two-tailed Student's *t*-test. Significance is indicated as follows: $p < 0.05$ (*), $p < 0.01$ (**), and n.s., not significant.

RPE-1 hTert, PLK1/3i

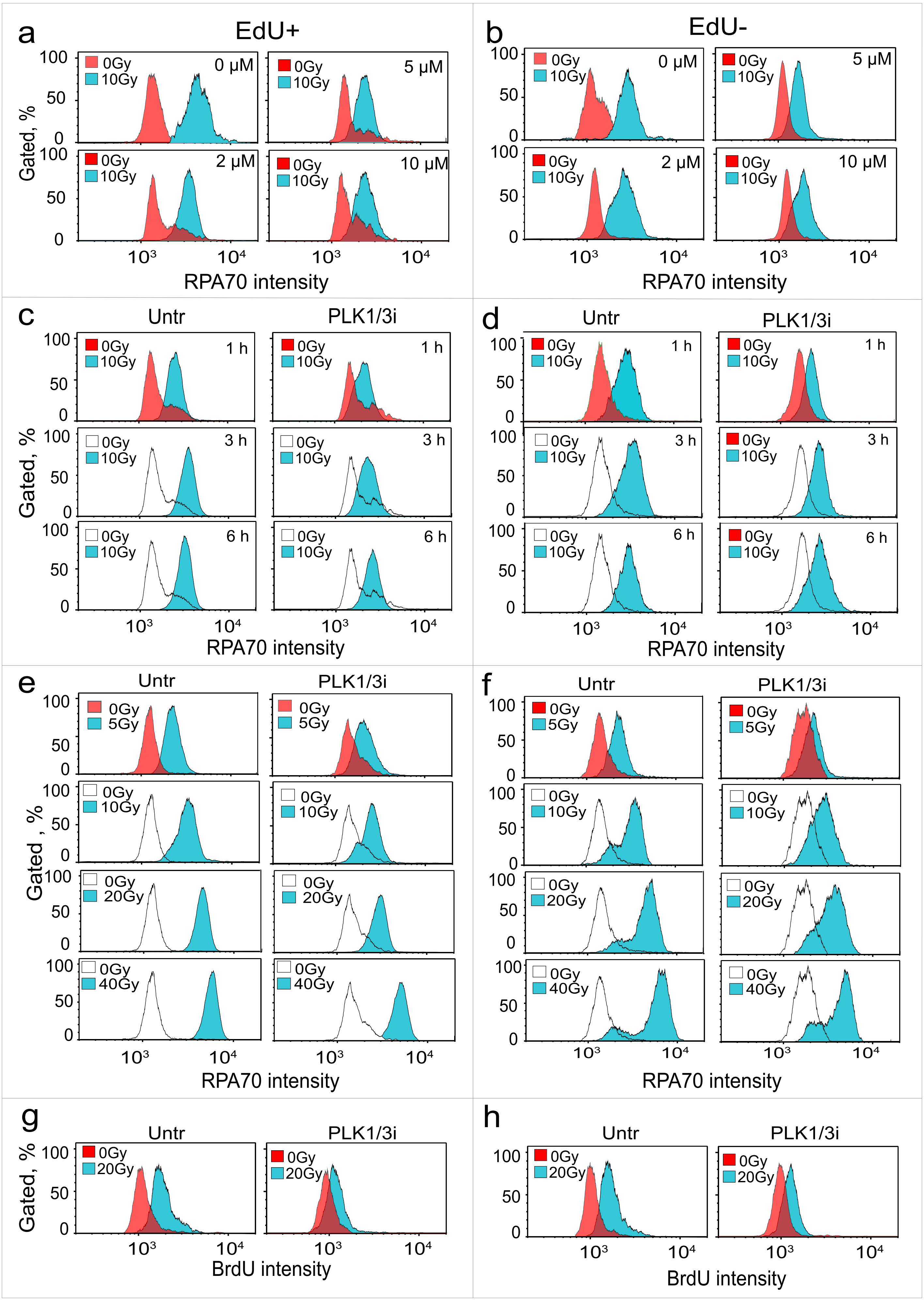

Figure S4, Pan et al.

Figure S4. PLK1/3 dual inhibition (PLK1/3i) suppresses resection in both EdU+ and EdU- G_2-phase RPE-1 hTert cells. RPE-1 hTert cells were pre-treated with PLK1/3i at the indicated concentrations for 1 h prior to IR exposure. (a–h) Representative flow cytometry plots from three independent experiments, corresponding to the quantification shown in Figure 3a–h, respectively. These plots illustrate the effects of PLK1/3i on RPA70 accumulation or BrdU exposure in EdU+ and EdU- G_2-phase cells under various treatment conditions. Approximately 10,000 cells were analyzed per sample in each experiment. Panels are representative of n = 3 independent biological replicates. No additional statistical analyses were performed for these representative datasets.

82-6 hTert, PLK1/3i

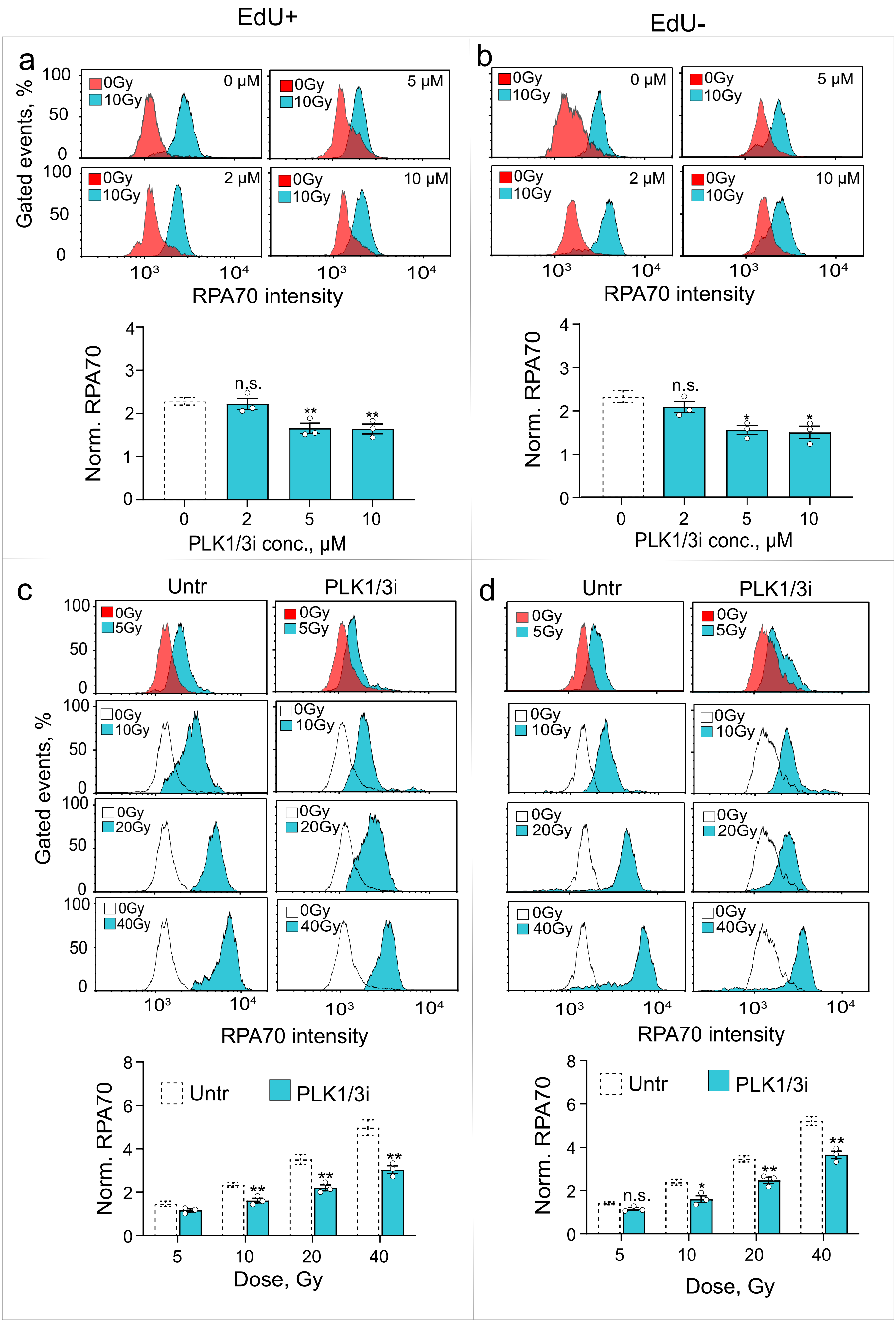

Figure S5, Pan et al.

Figure S5. PLK1/3i suppresses resection in both EdU+ and EdU- G_2-phase 82-6 hTert cells. 82-6 hTert cells were treated with the PLK1/3i and exposed to IR to assess the effects on resection. Chromatin-bound RPA70 was measured by flow cytometry 3 h post-IR as a marker of resection. (a) Effect of increasing concentrations of PLK1/3i (0, 2, 5, and 10 µM) on DNA end resection in EdU+ G_2-phase cells following 10 Gy IR. (b) Same as (a), but in EdU- G_2-phase cells. (c) RPA70 intensity in EdU+ G_2-phase cells treated with or without 5 µM PLK1/3i and exposed to increasing IR doses (0, 5, 10, 20, and 40 Gy). (d) Same as (c), but in EdU- G_2-phase cells. Experiments in Figure S5 were performed simultaneously with Figures S2 and S3 using the same experimental setup and shared controls. Representative flow cytometry plots shown in panels (a–d) are representative of three independent biological replicates. Bar plots show mean ± SEM from n = 3 independent biological replicates. Approximately 10,000 cells were analyzed per sample in each experiment. Statistical analyses were performed using an unpaired two-tailed Student's t-test. Significance is indicated as follows: $p < 0.05$ (*), $p < 0.01$ (**), and n.s., not significant.

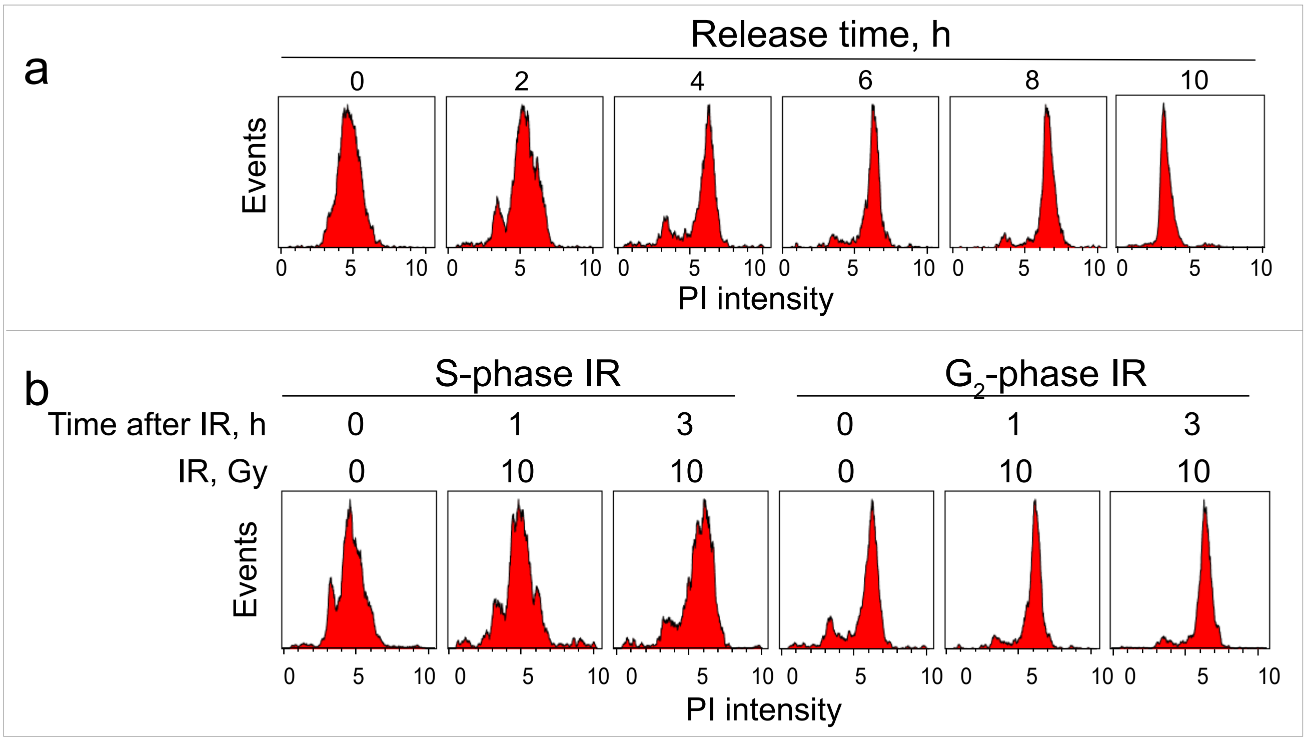

Figure S6 Pan et al.

Figure S6. Cell cycle distribution following STB treatment and IR exposure. RPE-1 hTert cells were synchronized using STB treatment and released to progress through the cell cycle as described in the Materials and Methods. (a) Flow cytometry analysis of DNA content at various time points following release from STB, corresponding to the experimental conditions in Figure 5g, lane 3-8. (b) Cell cycle profiles of cells collected 1 and 3 hours after exposure to 10 Gy IR, following release for 3 or 6 h from STB (corresponding to S- and G_2-phases, respectively). These data correspond to the conditions in Figure 5g, lane 9-14 and confirm successful synchronization and sample collection for PLK3 expression analysis. Experiments were repeated three times, and a representative plot is shown. Panels are representative of n = 3 independent biological replicates. Approximately 10,000 cells were analyzed per sample in each experiment.
