# Supplementary material for: Cell-cycle-dependent regulation of DNA end resection by PLK1 and PLK3 without CtIP level modulation
Source: iScience. 2026 Jul 1;29(7):116450. doi: 10.1016/j.isci.2026.116450 (PMC13355021; doi:10.1016/j.isci.2026.116450)

a.

Related to Figure 4a

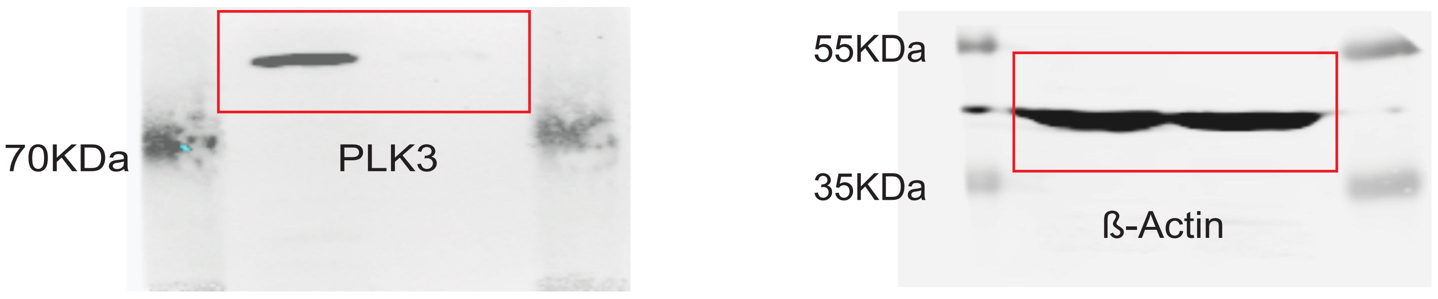

Exp 01, Presented in the main Figures as Figure 4a

b.

Related to Figure 4a

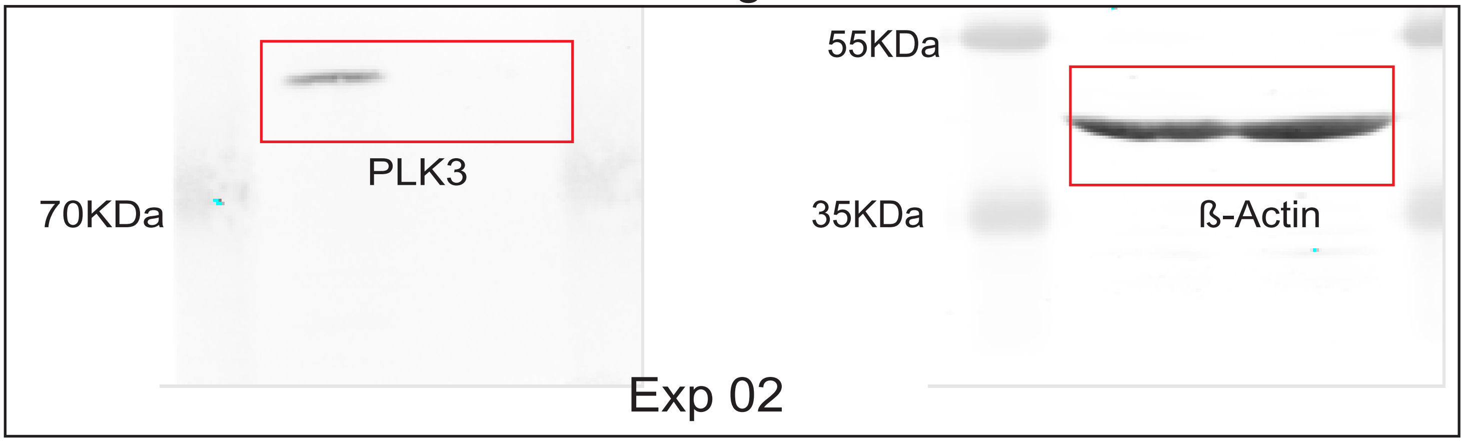

Exp 02

c.

Related to Figure 5g

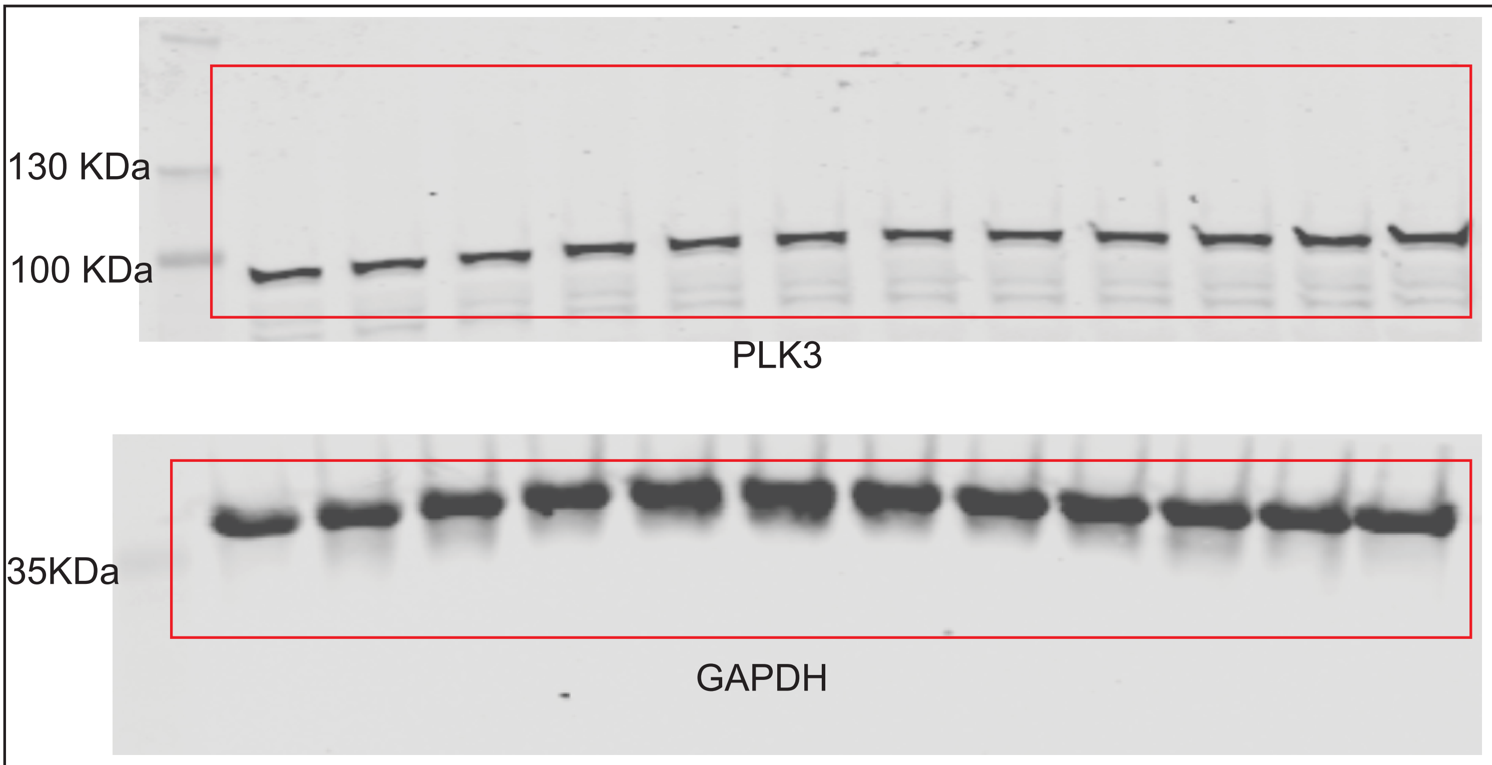

Exp 01, Presented in the main Figures as Figure 5g

d. Related to Figure 5g

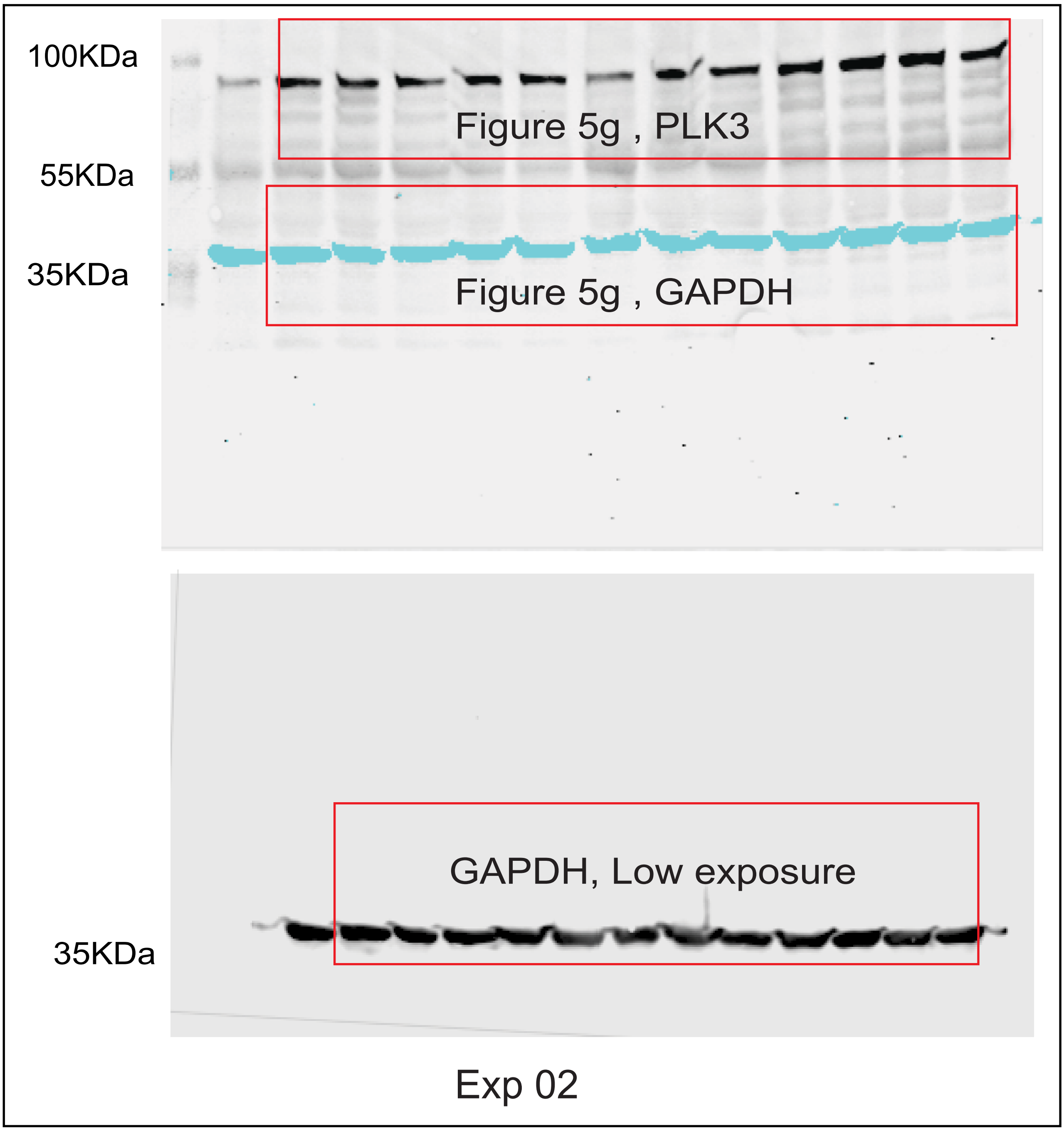

e. Related to Figure 6b

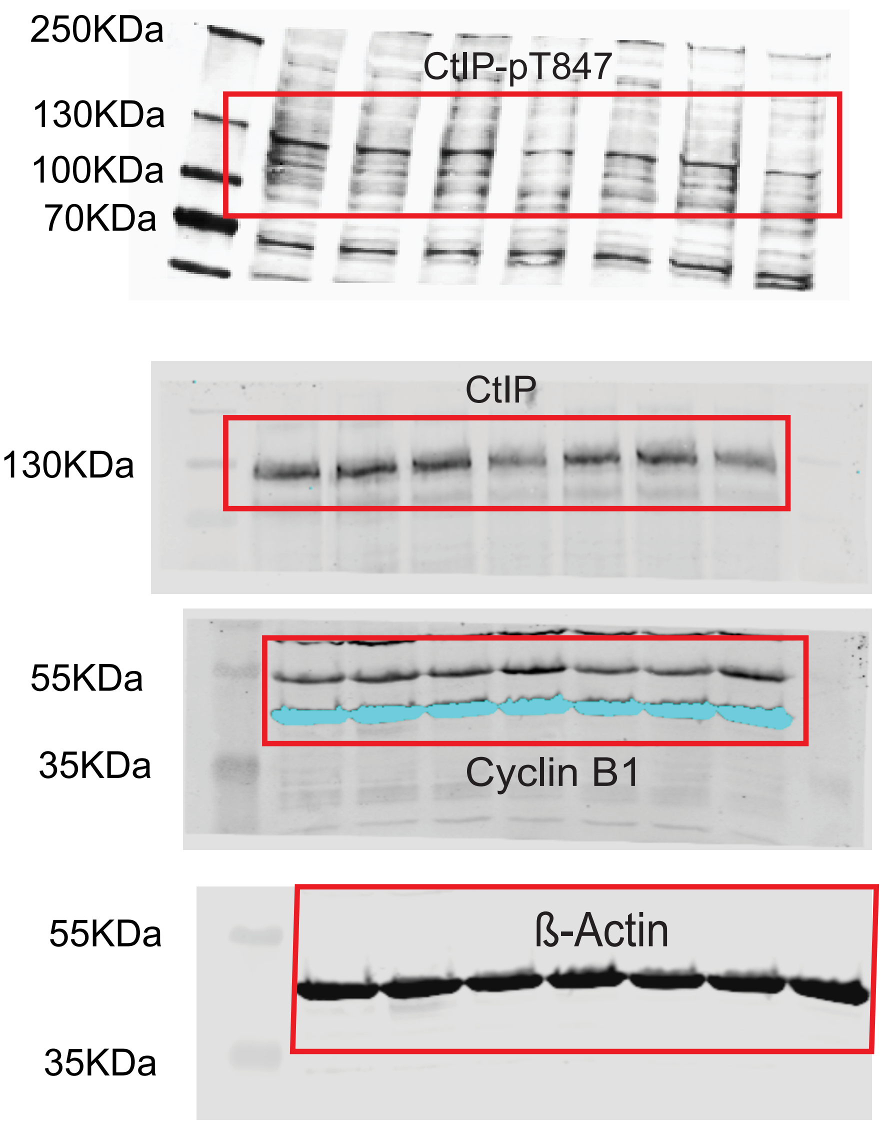

Exp 01, Presented in the main Figures as Figure 6b

f. Related to Figure 6b

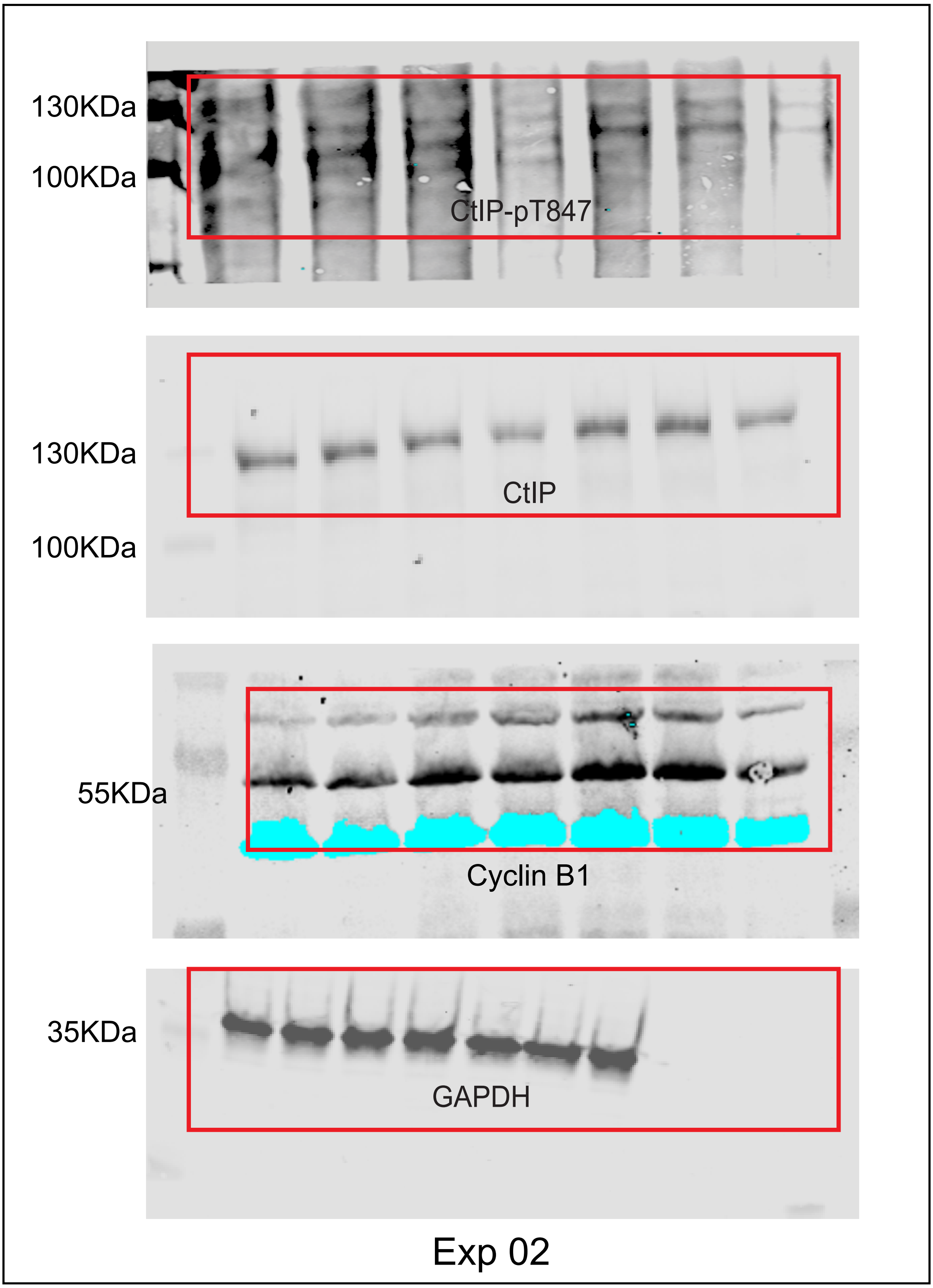

Supplement: Data S1. Original uncropped western blot images, related to Figures 4A, 5G, and 6B [file mmc2.pdf]
