# Supplementary material for: Cell-cycle-dependent regulation of DNA end resection by PLK1 and PLK3 without CtIP level modulation
Source: iScience. 2026 Jul 1;29(7):116450. doi: 10.1016/j.isci.2026.116450 (PMC13355021; doi:10.1016/j.isci.2026.116450)
Supplement: Data S2. Original uncropped microscopy images, related to Figure 7B [file mmc3.pdf]

a. Related to Figure 7b RPE-1hTert, untr. 0Gy, 3h

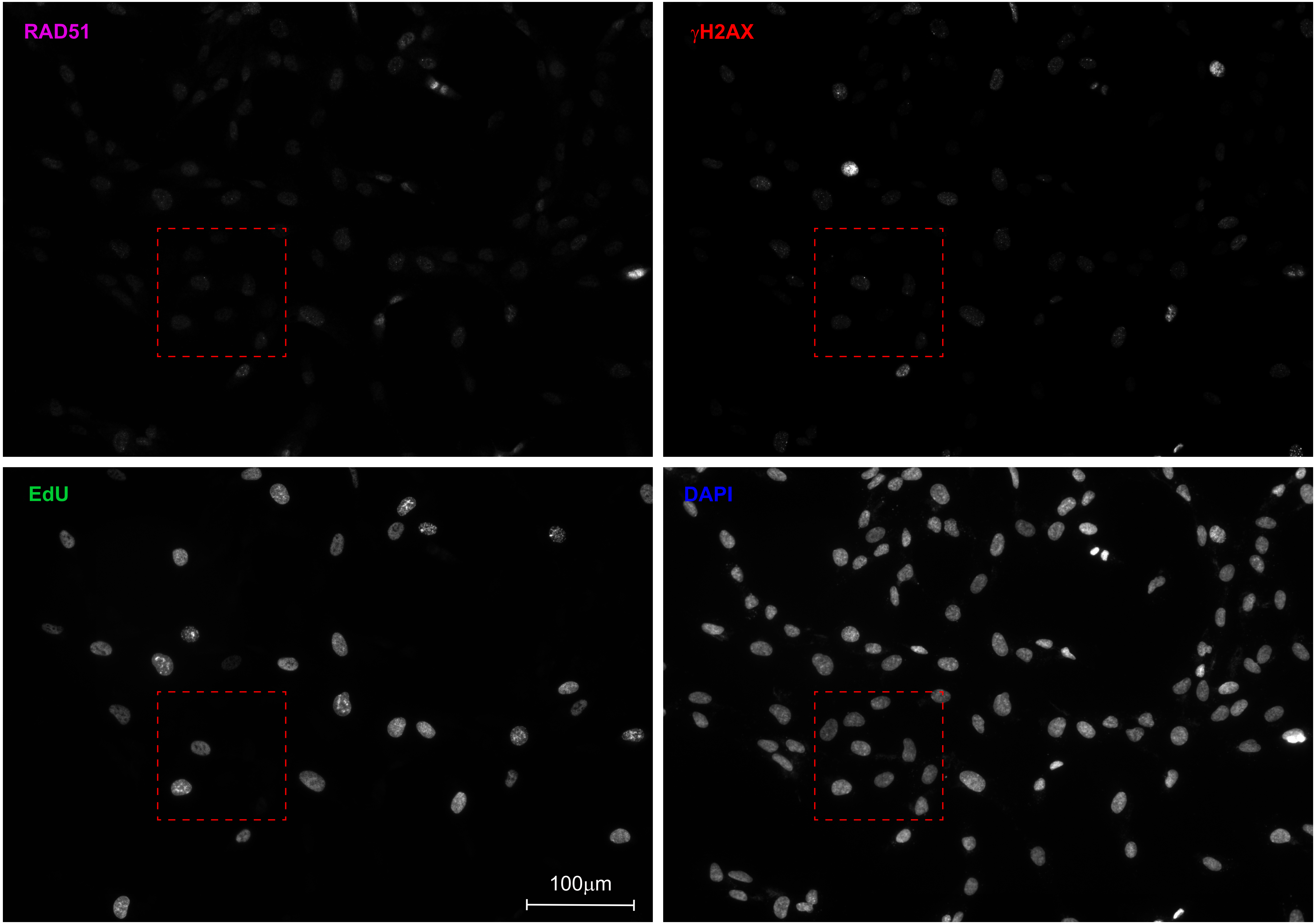

EXP1, presented in main Figures as Figure 7b

b. Related to Figure 7b,RPE-1hTert, untr. 2Gy, 3h

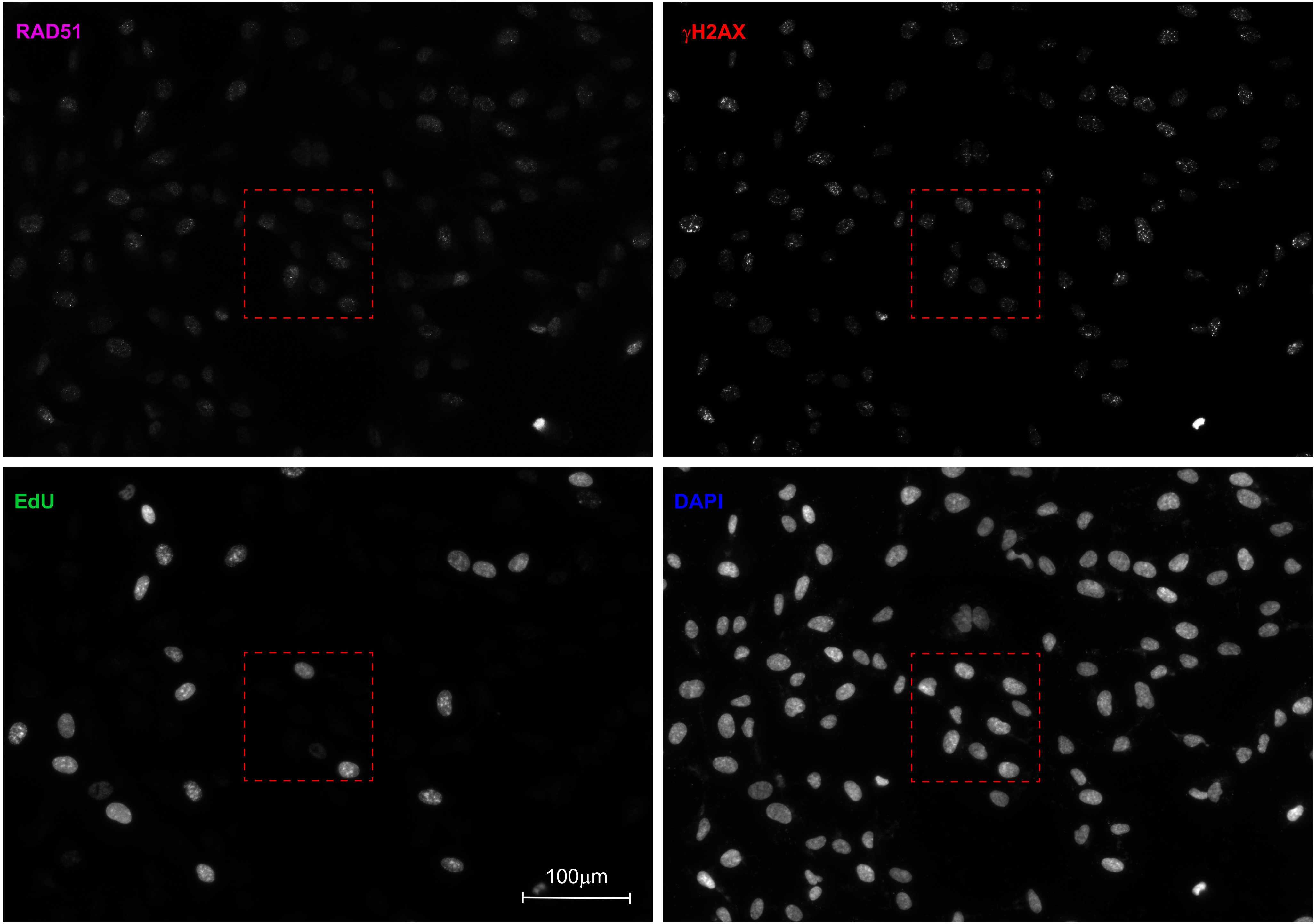

EXP1, presented in main Figures as Figure 7b

c. Related to Figure 7b, RPE-1hTert, untr. 0Gy, 3h

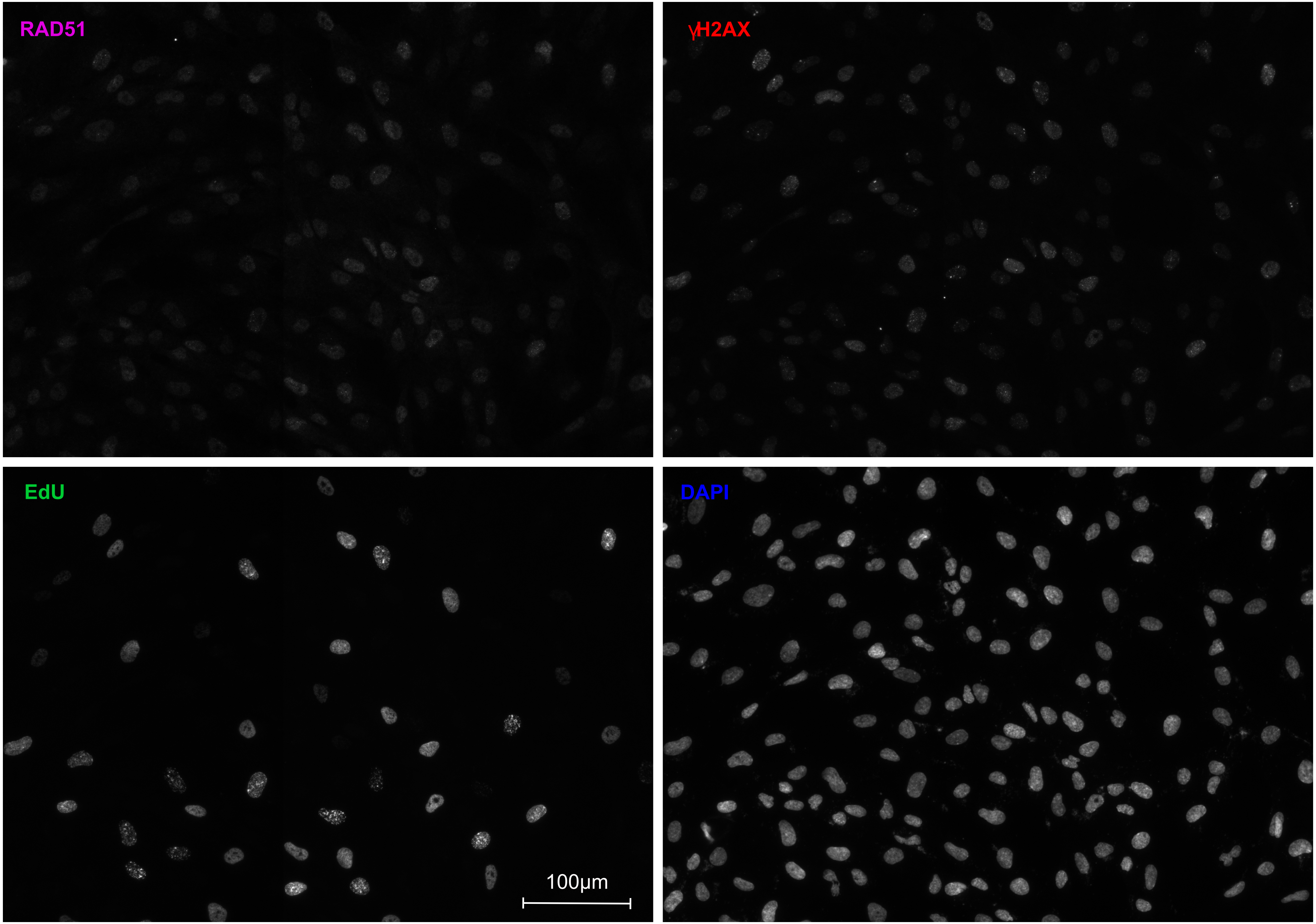

EXP 2

d. Related to Figure 7b, RPE-1 hTert, untr . 2Gy, 3h

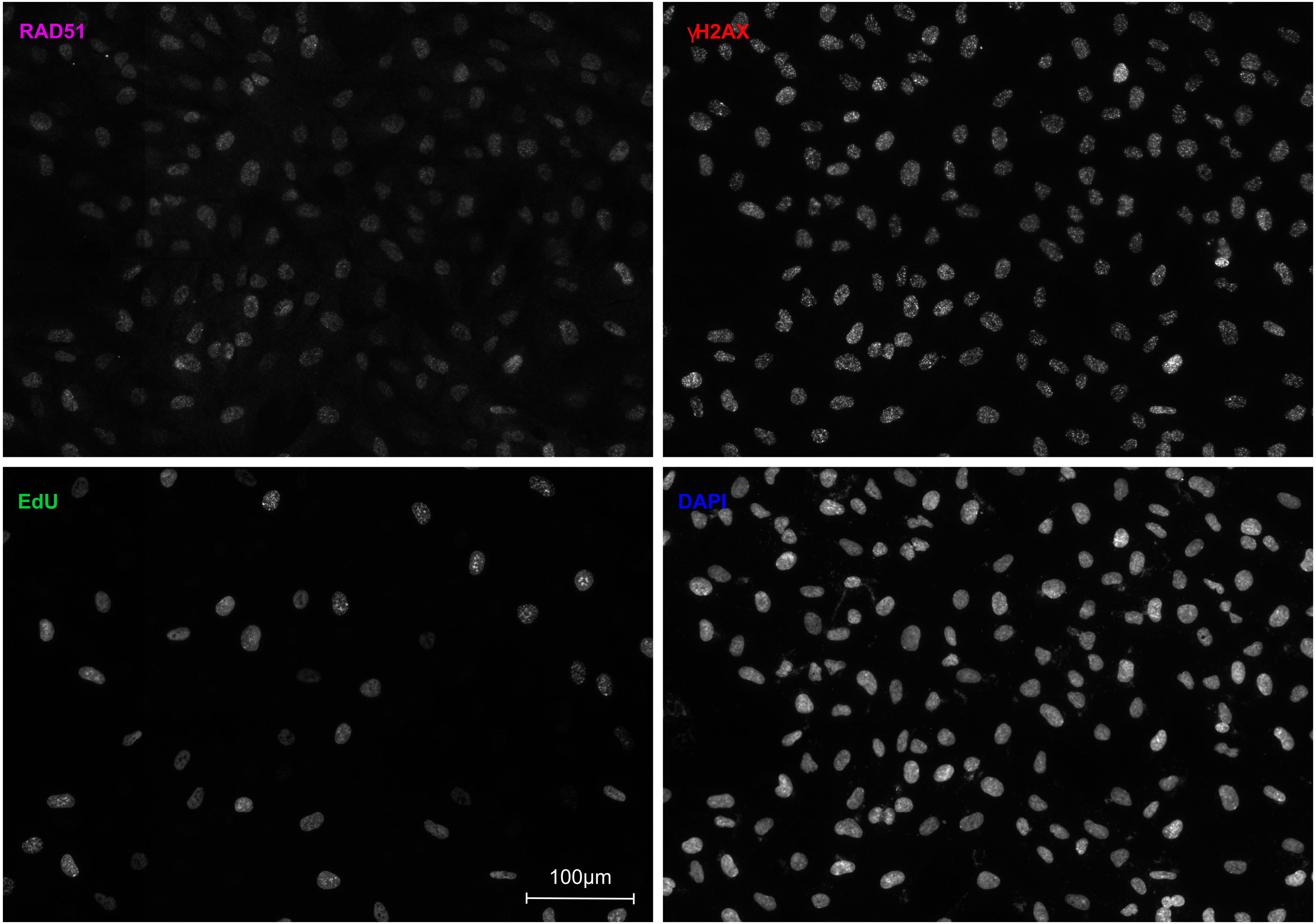

EXP2
